# Exosome‐Mediated Delivery of PROTACs for Targeted Protein Degradation in Cancer, Neurodegenerative, Infectious, and Inflammatory Diseases

**DOI:** 10.1111/jcmm.71297

**Published:** 2026-07-29

**Authors:** Sruti Bagchi Ghosh, Rumpa Banerjee, Hailah M. Almohaimeed, Amany I. Almars, Tahani Ahmad ALMatrafi, Fayez M. Saleh, Zuhair M. Mohammedsaleh, Kranti Kiran Reddy Ealla, Ajoy Kumer, Bikram Dhara

**Affiliations:** ^1^ Department of Pharmacy Calcutta Institute of Pharmaceutical Technology and Allied Health Sciences Howrah West Bengal India; ^2^ Department of Pharmacy Eminent College of Pharmaceutical Technology Barasat West Bengal India; ^3^ Department of Basic Science, College of Medicine Princess Nourah Bint Abdulrahman University Riyadh Saudi Arabia; ^4^ Department of Medical Laboratory Sciences, Faculty of Applied Medical Sciences King Abdulaziz University Jeddah Saudi Arabia; ^5^ Hematology Research Unit, King Fahd Medical Research Center King Abdulaziz University Jeddah Saudi Arabia; ^6^ Department of Anatomy, College of Medicine King Saud University Riyadh Saudi Arabia; ^7^ Department of Medical Microbiology, Faculty of Medicine University of Tabuk Tabuk Saudi Arabia; ^8^ Molecular Microbiology and Infectious Diseases Research Unit University of Tabuk Tabuk Saudi Arabia; ^9^ Department of Medical Laboratory Technology, Faculty of Applied Medical Sciences University of Tabuk Tabuk Saudi Arabia; ^10^ Department of Oral and Maxillofacial Pathology Malla Reddy Institute of Dental Sciences, Malla Reddy Vishwavidyapeeth Hyderabad Telangana India; ^11^ Department of Chemistry IUBAT‐International University of Business Agriculture & Technology Dhaka Bangladesh; ^12^ Department of Microbiology, Saveetha Medical College and Hospital Saveetha Institute of Medical and Technical Sciences Chennai Tamil Nadu India; ^13^ Department of Translational Medicine and Extracellular Vesicle Research ExoVesicle Biosciences Cambridge UK

**Keywords:** drug delivery, engineering strategies, exosomes, heterobifunctional, PROTACs, therapeutic applications

## Abstract

Proteolysis‐targeting chimeras (PROTACs) are heterobifunctional molecules that hijack the ubiquitin‐proteasome system to drive catalytic, sub‐stoichiometric degradation of disease‐associated proteins, offering a mechanistic advantage over occupancy‐driven inhibitors and access to ‘undruggable’ targets. However, their clinical translation is constrained by high molecular weight, poor solubility, low oral bioavailability, inefficient membrane permeability, nonspecific biodistribution, off‐target degradation, and the concentration‐dependent ‘hook effect.’ Exosomes, nanoscale extracellular vesicles with innate biocompatibility, low immunogenicity, prolonged circulation, and the ability to cross barriers such as the blood–brain barrier, offer a biologically integrated platform to overcome these limitations. This review traces the evolution of PROTAC technology, delineates the challenges of conventional delivery, and evaluates the rationale for exosomal encapsulation, including cargo protection, intracellular trafficking, endosomal escape, and release kinetics. We examine natural and engineered exosomes spanning source selection, active loading strategies, and surface functionalization for tissue‐specific homing and synthesize therapeutic applications across viral infections, cancer, neurodegenerative disorders, and inflammatory diseases. Proof‐of‐concept studies, such as camel milk‐derived exosomes delivering the BRD4‐targeting PROTAC ARV‐825, demonstrate enhanced permeability, lower IC_50_ values, and improved oral bioavailability. Finally, we discuss key hurdles to clinical translation: scalable production, purification, and standardization, and outline future directions for exosome‐mediated targeted protein degradation.

## 1 Introduction

Targeted protein degradation has emerged as a transformative strategy in modern drug discovery, offering a powerful alternative to traditional small‐molecule inhibition. Among the most advanced approaches within this paradigm are PROTACs, a class of heterobifunctional molecules designed to induce selective degradation of disease‐associated proteins through the ubiquitin‐proteasome system (UPS) [1]. Unlike conventional inhibitors that rely on sustained target occupancy to suppress protein function, PROTACs operate catalytically by eliminating the target protein itself, thereby achieving prolonged pharmacological effects at lower doses.

**FIGURE 1:**
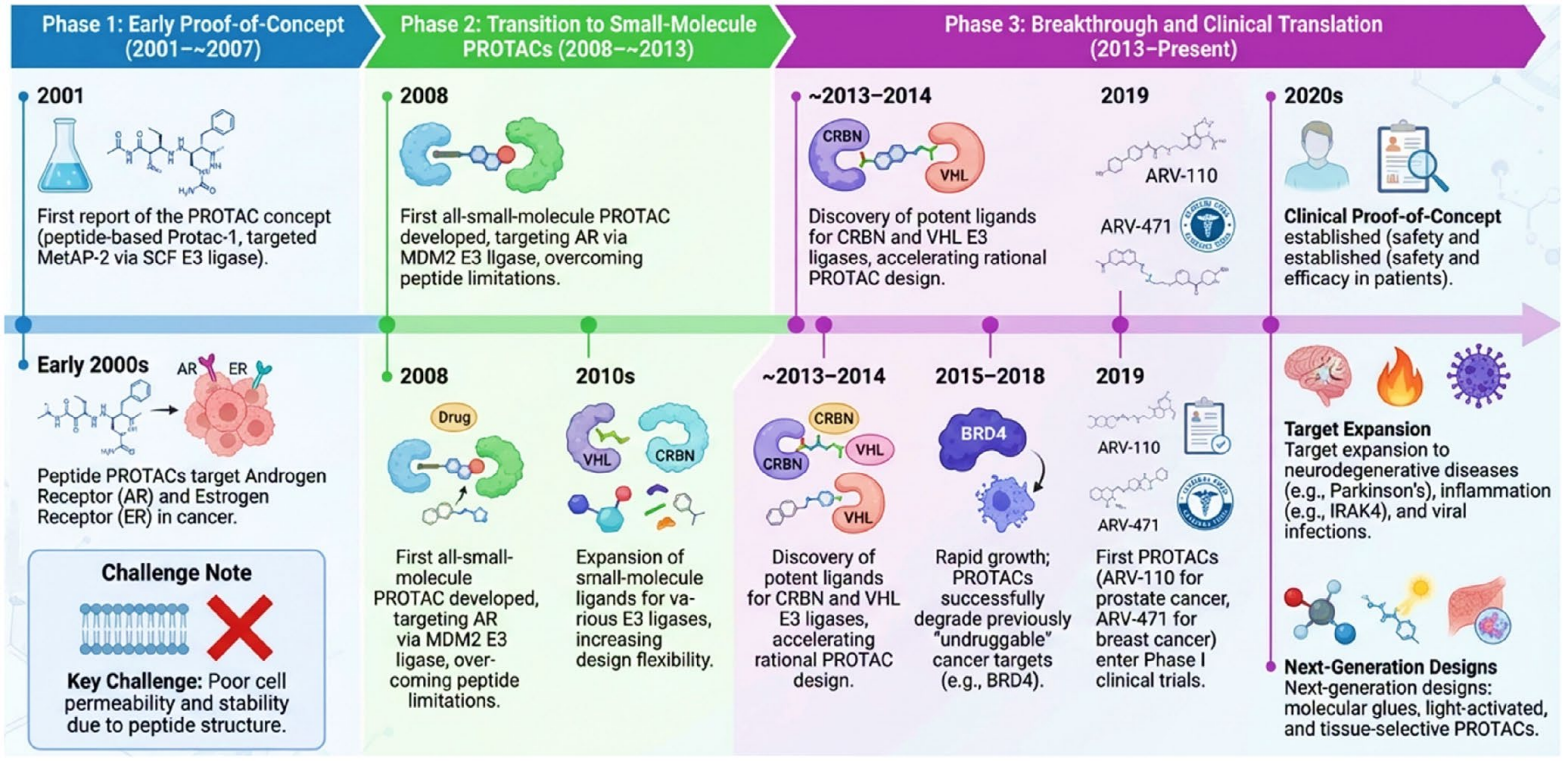
Timeline of development of PROTACs.

The conceptual foundation of PROTAC technology was first introduced in 2001, when chimeric molecules capable of recruiting target proteins to E3 ubiquitin ligases were proposed to hijack the cell's endogenous degradation machinery. Early PROTAC designs employed peptide‐based ligands and were limited by poor cellular permeability and metabolic instability. Subsequent advances led to the development of fully small‐molecule PROTACs, with the incorporation of ligands for E3 ligases such as MDM2, inhibitor of apoptosis proteins (IAPs), von Hippel–Lindau (VHL), and cereblon (CRBN), significantly improving their drug‐like properties [1]. By 2019, the clinical potential of PROTACs was underscored by the entry of multiple candidates into human clinical trials, marking a major milestone in the field (Figure 1). Structurally, PROTACs consist of three essential components: a ligand that binds the protein of interest (POI), a ligand that recruits an E3 ubiquitin ligase, and a linker that connects the two. Upon binding, PROTACs facilitate the formation of a ternary complex between the POI and the E3 ligase, promoting ubiquitination of the target protein. This ubiquitin tagging serves as a signal for recognition by the 26S proteasome, resulting in selective degradation of the POI [2]. Importantly, because PROTACs are released after target degradation, a single PROTAC molecule can trigger multiple rounds of protein elimination, contributing to their catalytic efficiency.

PROTACs offer several advantages over traditional small‐molecule inhibitors; these include the ability to target non‐enzymatic and scaffolding proteins previously considered ‘undruggable,’ reduced susceptibility to resistance caused by target overexpression or mutation, and sustained biological effects even after compound clearance [3]. Additionally, PROTAC‐mediated degradation removes all functions of the target protein, including non‐catalytic roles, which cannot be achieved by active‐site inhibition alone. These attributes have driven intense interest in the application of PROTACs for cancer, viral infections, inflammatory disorders, and neurodegenerative diseases [4].

**FIGURE 2:**
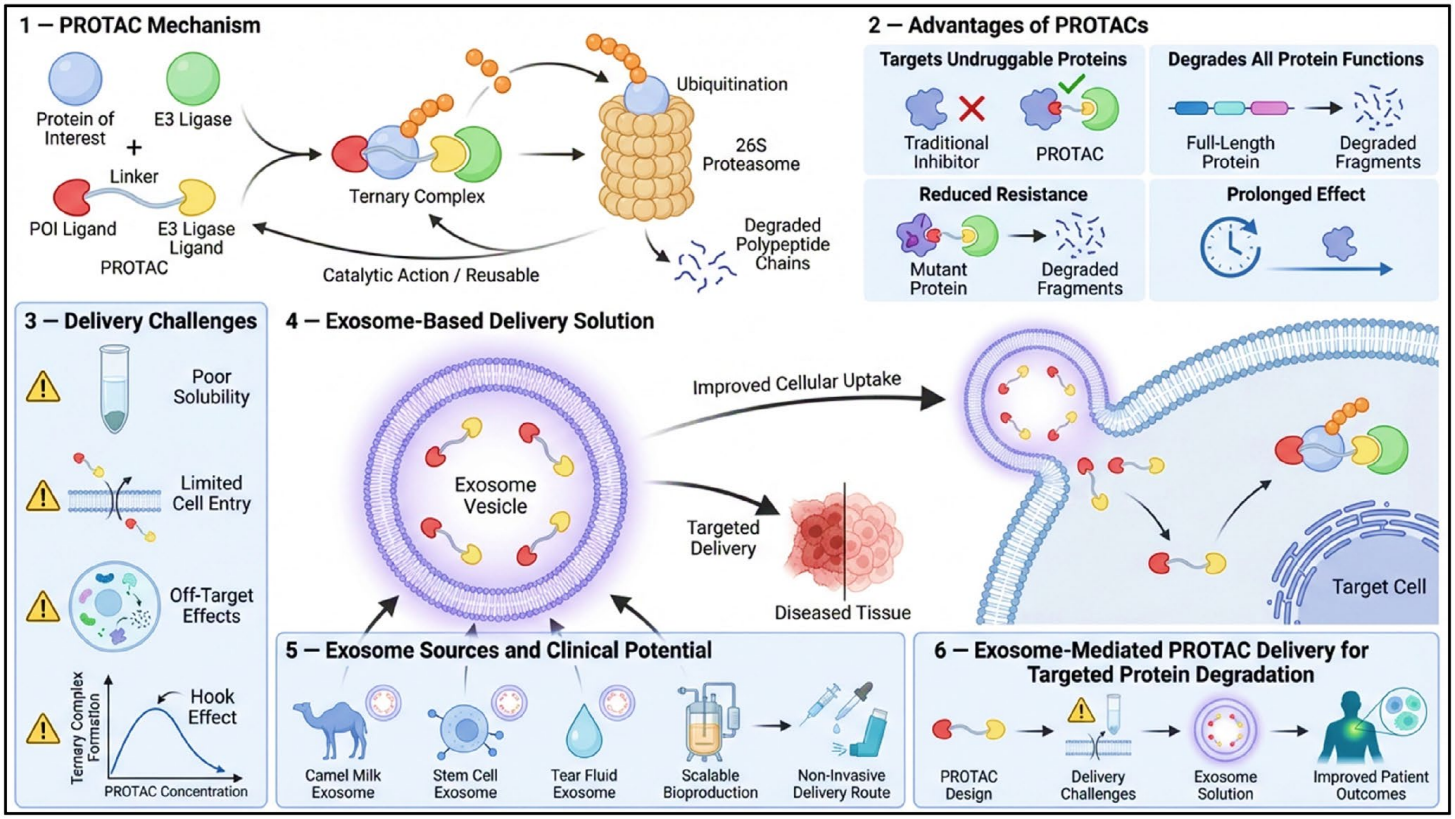
Perspective of Exosome based delivery of PROTACs.

Despite all these promising features, the clinical translation of PROTACs remains challenged by significant delivery‐related limitations. PROTACs are typically large and structurally complex molecules, often exhibiting poor aqueous solubility, limited membrane permeability, and suboptimal bioavailability. Furthermore, nonspecific tissue distribution and dependence on the UPS can lead to off‐target protein degradation and systemic toxicity [5]. These issues are compounded by dose‐dependent effects such as the ‘hook effect,’ wherein excessive PROTAC concentrations reduce productive ternary complex formation, ultimately diminishing degradation efficiency [3]. To overcome these barriers, novel drug delivery strategies are being actively explored, among which exosome‐based delivery has gained considerable attention. Exosomes are nanosized extracellular vesicles (30–150 nm) secreted by most cell types and play a fundamental role in intercellular communication by transporting proteins, lipids, and nucleic acids [6]. Their intrinsic biocompatibility, low immunogenicity, prolonged circulation time, and ability to cross biological barriers such as the BBB position exosomes as highly attractive carriers for therapeutic payloads, including PROTACs (Figure 2) [7, 8, 9].

Recent studies demonstrate that loading PROTACs into exosomes can markedly improve their stability, cellular uptake, and tissue specificity, while simultaneously reducing off‐target toxicity [10, 11]. Exosome‐mediated delivery systems derived from diverse biological sources, including camel milk, stem cells, and tear fluid, further expand the translational potential of this approach by enabling non‐invasive administration routes and scalable production strategies [12, 13, 14] (Figure 2). Collectively, these advances suggest that exosome‐based PROTAC delivery may represent a critical step toward realizing the full therapeutic potential of targeted protein degradation.

## 2 Limitations of Conventional PROTAC Delivery

PROTACs have fundamentally reshaped the landscape of targeted therapeutics by introducing a degradation‐based mechanism rather than functional inhibition of disease‐associated proteins. Despite their conceptual elegance and demonstrated preclinical efficacy, the clinical advancement of PROTACs has been considerably slowed by challenges associated with their delivery. These limitations arise from a combination of unfavourable physicochemical properties, biological barriers to intracellular access, nonspecific biodistribution, and intrinsic pharmacodynamic complexities. Understanding these constraints is essential for appreciating why advanced delivery strategies, such as exosome‐based systems, are increasingly being explored to unlock the full therapeutic potential of PROTAC technology. One of the primary barriers to effective PROTAC delivery lies in their physicochemical characteristics. PROTACs are structurally complex heterobifunctional molecules composed of two ligands linked together by a flexible chemical spacer. This design often results in compounds with high molecular weights, frequently exceeding 800–1000 Da, and substantial polar surface areas [1]. Such properties deviate markedly from classical drug‐likeness criteria and have direct implications for solubility, stability, and absorption. Many PROTACs exhibit poor aqueous solubility, which limits their formulation options and reduces oral bioavailability. As a result, a significant fraction of administered drug may fail to reach systemic circulation in an active form, thereby diminishing therapeutic efficacy. Although certain cereblon‐based PROTACs have demonstrated partial oral bioavailability, others, particularly those recruiting the VHL E3 ligase, often require intravenous administration due to rapid clearance and inadequate absorption [5]. In addition to solubility issues, PROTACs are susceptible to metabolic degradation, particularly by hepatic enzymes, which further compromises their pharmacokinetic profile. Rapid metabolism can shorten plasma half‐life, necessitating higher or more frequent dosing to maintain therapeutic concentrations. This not only complicates dosing regimens but also increases systemic exposure, thereby elevating the risk of toxicity. These pharmacokinetic limitations highlight the inadequacy of conventional formulation approaches for PROTACs and underscore the need for delivery systems capable of protecting these molecules from premature degradation [3] Another major challenge is the inefficient cellular uptake of PROTACs. For PROTACs to exert their biological effect, they must gain access to the intracellular environment, where the UPs operate. However, the large size, high polarity, and flexible nature of PROTAC molecules severely limit their ability to cross cellular membranes via passive diffusion. Unlike traditional small‐molecule inhibitors, which often exploit lipophilicity to penetrate cells, PROTACs frequently display poor membrane permeability, resulting in insufficient intracellular concentrations even when extracellular levels are high [4]. This problem is further compounded in solid tumours and fibrotic or inflamed tissues, where dense extracellular matrices and abnormal vasculature impede drug penetration. Consequently, achieving effective intracellular concentrations may require elevated systemic doses, increasing the likelihood of adverse effects in non‐target tissues. The reliance of PROTACs on the UPS introduces additional safety concerns related to off‐target protein degradation. The proteasome is a ubiquitous and essential cellular machinery responsible for maintaining protein homeostasis in both healthy and diseased cells. Because PROTACs do not inherently distinguish between pathological and physiological expression of a target protein, systemic administration can lead to unintended degradation of proteins in healthy tissues [1], this off‐target activity can disrupt critical cellular processes and result in cytotoxicity, particularly when the targeted protein performs essential functions across multiple tissues. Unlike classical inhibitors, which may allow partial inhibition without complete functional loss, PROTAC‐mediated degradation eliminates all functions of the protein, including non‐enzymatic and scaffolding roles. This irreversible removal can amplify adverse effects and significantly narrow the therapeutic window.

Closely related to off‐target toxicity is the lack of tissue and cell‐type specificity associated with conventional PROTAC delivery. Following systemic administration, PROTACs distribute broadly throughout the body, often accumulating in organs involved in drug metabolism and clearance, such as the liver and kidneys. This nonspecific biodistribution reduces the fraction of drug reaching the intended disease site and increases exposure of healthy tissues to potentially harmful effects [5]. In diseases such as cancer, where tumour heterogeneity and microenvironmental complexity necessitate precise spatial control of drug action, the absence of intrinsic targeting mechanisms severely limits therapeutic effectiveness. Similar challenges are encountered in neurological disorders, where the BBB restricts access of large molecules, and in inflammatory diseases, where localized delivery is often critical for minimizing systemic immunosuppression.

**FIGURE 3:**
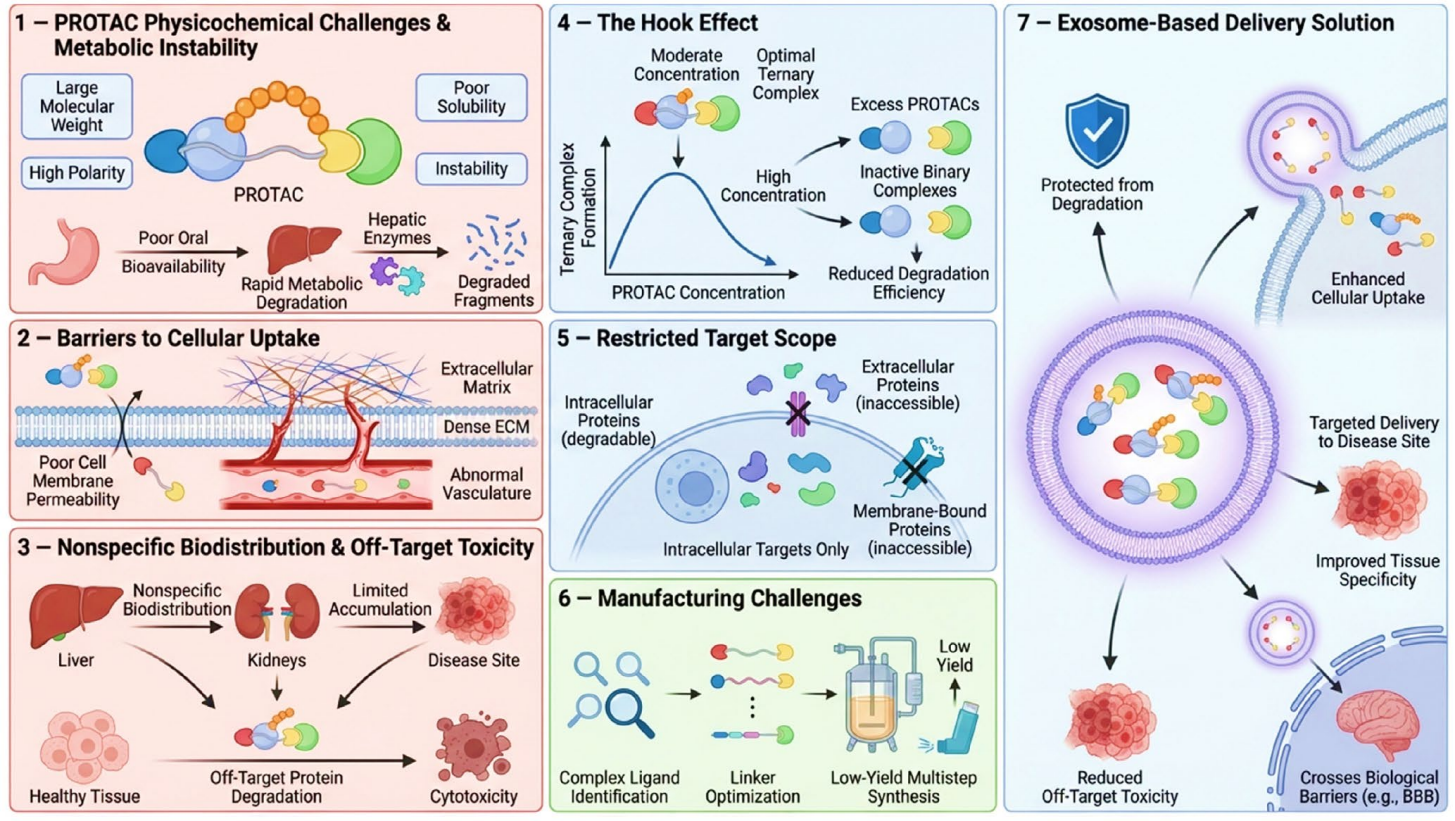
Limitations of conventional PROTAC delivery.

Pharmacodynamic complexity further complicates conventional PROTAC delivery, particularly due to the phenomenon known as the ‘hook effect.’ PROTAC activity depends on the formation of a productive ternary complex between the protein of interest and the recruited E3 ligase. At optimal concentrations, PROTACs efficiently promote this interaction, leading to ubiquitination and subsequent proteasomal degradation. However, at higher concentrations, excess PROTAC molecules can saturate the binding sites of either the target protein or the E3 ligase independently, favouring the formation of inactive binary complexes rather than the functional ternary complex [3]. This results in a paradoxical decrease in degradation efficiency at elevated doses. The hook effect complicates dose optimization and demands precise control over drug exposure, which is difficult to achieve with conventional delivery methods that lack sustained and controlled release properties. Another important limitation of traditional PROTAC strategies is their restricted target scope. Conventional PROTACs primarily act on intracellular proteins, as they depend on the cytosolic proteasome for degradation. Consequently, extracellular proteins and membrane‐bound proteins that are not efficiently internalized remain largely inaccessible [15]. This constraint excludes a broad class of clinically relevant targets involved in extracellular signalling, immune modulation, and host‐pathogen interactions. While emerging approaches such as lysosome‐targeting chimeras aim to address this gap, conventional PROTAC delivery systems remain limited in their ability to expand the degradable proteome. Challenges related to PROTAC design and manufacturing indirectly exacerbate delivery issues. The identification of suitable ligands for both the target protein and the E3 ligase is a complex and resource‐intensive process. Linker length, flexibility, and chemical composition must be carefully optimized, as minor modifications can dramatically influence ternary complex stability and degradation efficiency [4]. From a production standpoint, PROTAC synthesis often involves multistep chemical reactions with low yields, increasing costs and limiting scalability. These factors complicate formulation development and hinder the translation of promising candidates into clinically viable therapies. While PROTACs represent a powerful and innovative therapeutic modality, their conventional delivery is hampered by significant physicochemical, biological, and pharmacological challenges. Poor solubility limited cellular uptake, nonspecific biodistribution, off‐target degradation, dose‐dependent inefficiencies, and manufacturing complexities collectively restrict their clinical applicability. These limitations strongly justify the exploration of advanced delivery platforms, such as exosome‐based systems, which offer the potential to enhance stability, improve targeting, and reduce systemic toxicity, thereby enabling the broader clinical translation of PROTAC‐based therapies (Figure 3).

## 3 Rationale for Exosome‐Based PROTAC Delivery

Polymeric nanoparticles, liposomes, dendrimers, inorganic nanoparticles, antibody‐drug conjugates and prodrug‐based systems have been investigated to overcome limitations on pharmacokinetics and intracellular delivery of PROTAC therapeutics. Some of these delivery platforms successfully showed enhancements in solubility, circulation time in vivo, tumour targeting efficiency, cellular internalization and other attributes. Despite successes, there are many limitations in synthetic nanocarriers due to quick uptake by the mononuclear phagocyte system (MPS), immunogenicity, penetration across biological barriers, formulation stability and off‐target toxicity etc. Intracellular trafficking of PROTACs within target cells and cytoplasmic release are one of the main difficulties in conventional nanoparticle systems to bring PROTAC into the cell cytoplasm. Exosomes are emerging to be biological nanocarriers with innate biocompatibility, negligible immunogenicity, biological membrane constitution and inherent capability of intercellular communication; therefore, a potential platform for PROTAC delivery [3].

**FIGURE 4:**
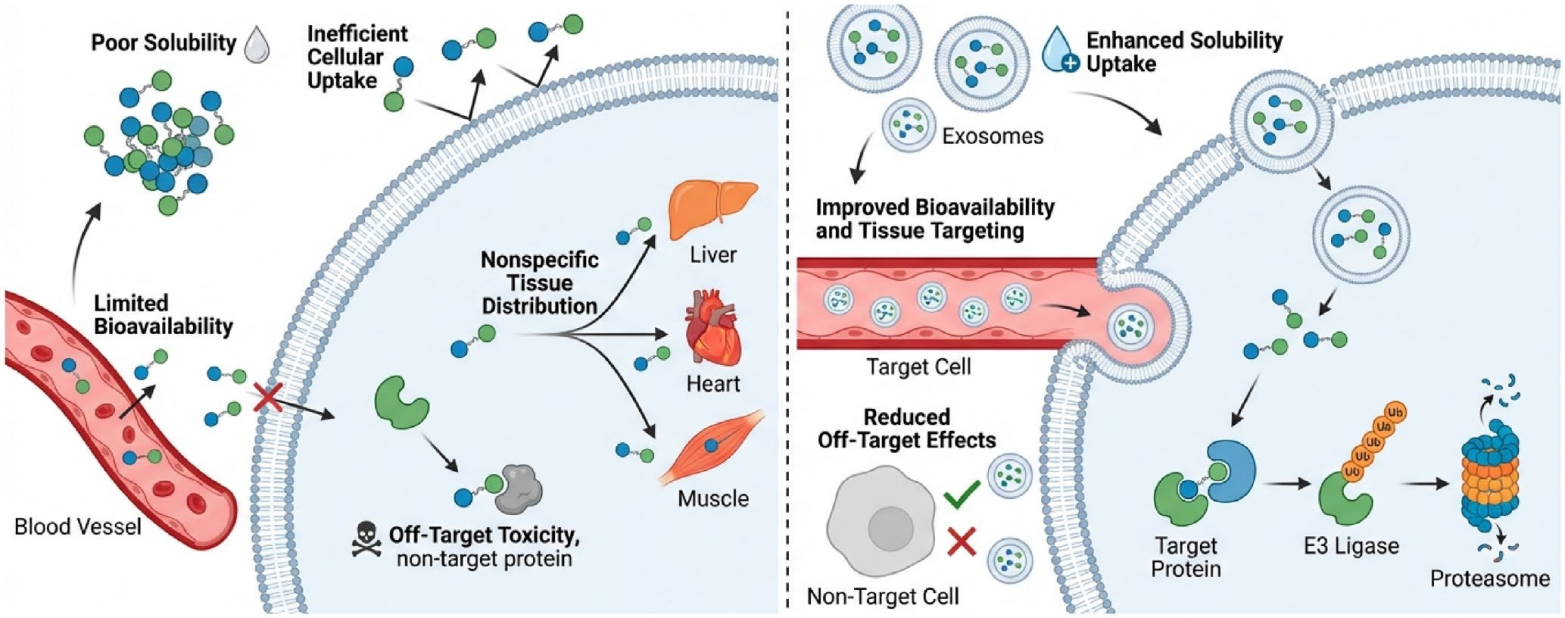
PROTACs vs. exosome based PROTACs delivery.

Although PROTACs have emerged as a powerful therapeutic modality for selective degradation of disease‐associated proteins, their clinical translation has been hindered primarily by delivery‐related challenges. Conventional delivery approaches fail to adequately address the poor solubility, limited bioavailability, inefficient cellular uptake, nonspecific tissue distribution, and off‐target toxicity inherent to many PROTAC molecules [3]. These limitations have driven increasing interest in biologically inspired delivery systems capable of enhancing the pharmacokinetic and pharmacodynamic performance of PROTACs. Among the various nanocarrier platforms explored, exosomes have gained considerable attention due to their intrinsic biological compatibility and natural role in intercellular cargo transport (Figure 4).

Exosomes are nanosized extracellular vesicles, typically ranging from 34 to 150 nm in diameter, that are secreted by nearly all cell types under physiological and pathological conditions. They play a critical role in cell‐to‐cell communication by transferring proteins, lipids, and nucleic acids between cells, thereby influencing a wide range of biological processes [16, 17] Their endogenous origin confers several advantages over synthetic nanocarriers, including low immunogenicity, minimal toxicity, and high biocompatibility, making them particularly attractive for therapeutic delivery applications [11, 18, 19, 20]. Importantly, exosomes possess inherent stability in biological fluids and can protect encapsulated cargo from enzymatic degradation, which is highly relevant for structurally complex molecules such as PROTACs. One of the key rationales for employing exosomes as PROTAC delivery vehicles lies in their ability to improve drug stability and bioavailability. PROTACs are often prone to rapid degradation and clearance following systemic administration, resulting in reduced therapeutic exposure. Encapsulation within exosomes provides a protective lipid bilayer environment that shields PROTACs from premature metabolic breakdown, thereby prolonging circulation time and enhancing systemic availability [11, 18]. This protective effect is particularly beneficial for orally administered formulations, where PROTACs are otherwise vulnerable to harsh gastrointestinal conditions and first‐pass metabolism. Milk‐derived exosomes, including bovine and camel milk exosomes, have demonstrated remarkable stability in the gastrointestinal tract and have been shown to facilitate oral delivery of bioactive compounds [6, 13].

Exosome‐mediated delivery also addresses one of the most critical barriers in PROTAC therapeutics: inefficient cellular uptake. Due to their large molecular size and high polarity, PROTACs exhibit limited membrane permeability, which restricts their intracellular access to the ubiquitin–proteasome system. Exosomes naturally interact with recipient cells through membrane fusion, endocytosis, or receptor‐mediated uptake, enabling efficient intracellular delivery of their cargo [7]. By exploiting these physiological uptake mechanisms, exosomes can significantly enhance the intracellular concentration of PROTACs, thereby improving target engagement and degradation efficiency while reducing the need for high systemic doses. Another compelling rationale for exosome‐based PROTAC delivery is the potential for improved targeting specificity and reduced off‐target toxicity. Conventional PROTACs distribute non‐specifically across tissues, leading to unintended protein degradation in healthy cells. Exosomes, however, can be engineered to display specific surface ligands or membrane proteins that promote selective homing to target tissues or cell types [8, 21]. This targeting capability is particularly advantageous in diseases such as cancer and viral infections, where selective delivery to diseased cells can substantially improve therapeutic efficacy while minimizing systemic toxicity. Moreover, the endogenous targeting properties of exosomes derived from specific cell sources further enhance tissue selectivity, thereby narrowing the therapeutic window and improving safety profiles. The ability of exosomes to traverse biological barriers represents another major advantage over conventional delivery systems. Exosomes have been shown to cross challenging physiological barriers, including the BBB, making them uniquely suited for delivering therapeutics to the central nervous system [22] This property is especially relevant for PROTAC‐based interventions in neurodegenerative disorders, where effective delivery across the BBB remains a major obstacle. Similarly, exosomes derived from tear fluid have emerged as promising non‐invasive delivery vehicles for ocular and neurological applications, offering direct access to sensitive tissues while minimizing immune activation [14, 17].

### 3.1 Intracellular Trafficking, Endosomal Escape, and Release Kinetics of Exosome‐Delivered PROTACs

The intracellular trafficking of exosomes determines the effectiveness of the PROTAC delivery system. After exosome uptake into the cells (through clathrin‐mediated endocytosis, caveolin‐dependent internalization, macropinocytosis or direct membrane fusion), exosomes enter an early endosomal compartment. A significant hurdle in intracellular drug delivery is the endosomal confinement and subsequent lysosomal degradation of entrapped therapeutics. As PROTACs are only functional in the cytoplasm, they need to release from the endosome to interact with the cytosolic ubiquitin‐proteasome system for formation of the ternary complex and subsequently Ub and degrade the targeted protein. The various reported mechanisms for exosomal release from endosomal compartments, such as pH‐dependent membrane destabilization, lipid fusion mediated release, proton sponge effect, incorporation of fusogenic peptides and insertion of engineered membrane protein. These events facilitate endosomal membrane rupture and cytosolic release of loaded PROTAC, thus leading to elevated intracellular bioavailability and degradative activity. The rate of intracellular release of PROTAC also affects the pharmacodynamics. Effective degradation requires sustained intracellular concentration. Too fast burst release will lead to a higher concentration of intracellular PROTAC, hence increasing the possibility of the hook effect and decreasing the degradation rate. Controlled, steady release may result in optimal concentration of the protein in the cells and facilitate prolonged, effective protein catalysis. The lipid bilayer structure of the exosome acts as a biologically friendly reservoir that enables gradual release of the PROTAC into the cytoplasm, meanwhile protecting it from being rapidly degraded by enzymes.

**FIGURE 5:**
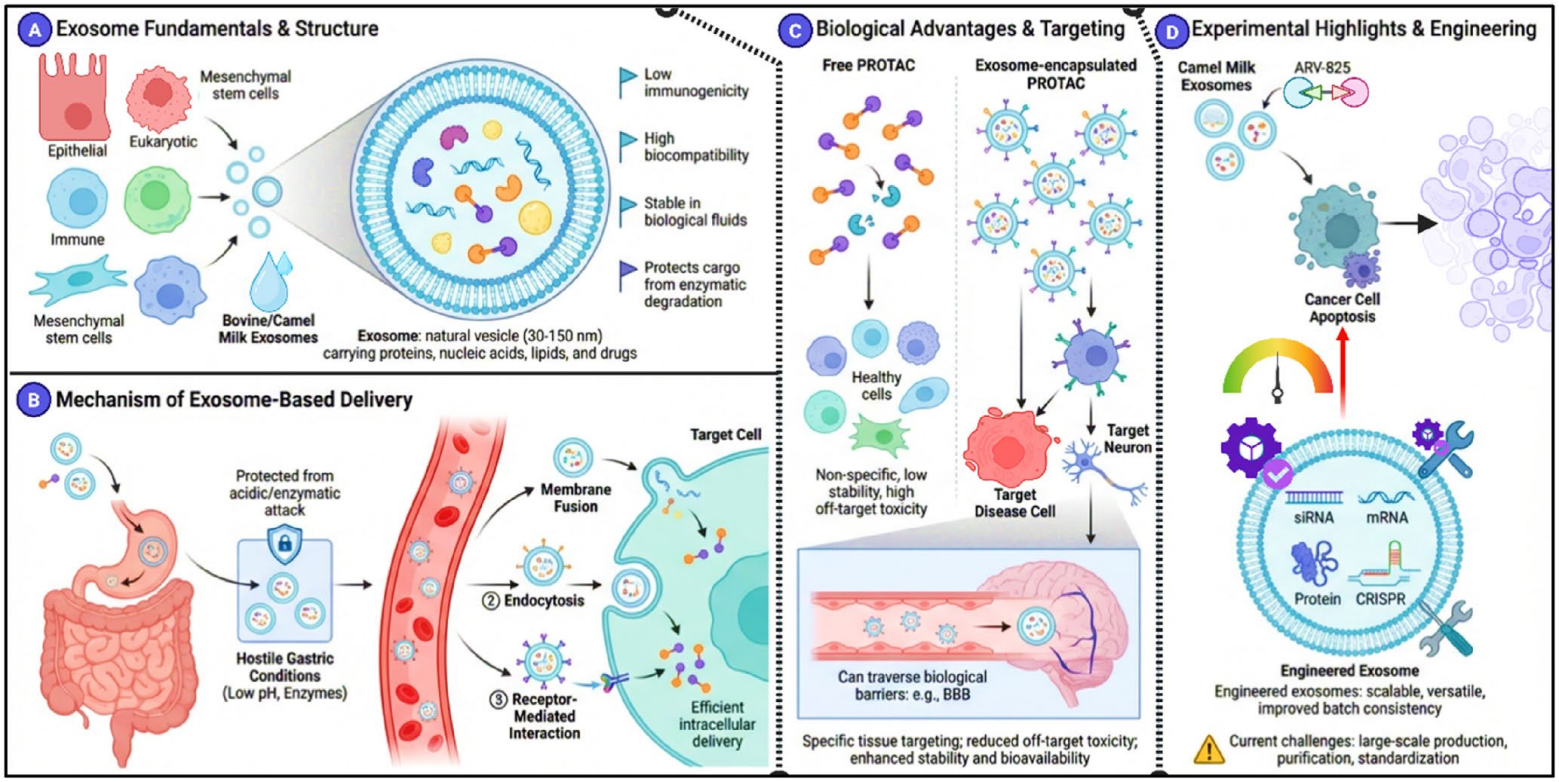
Rationale behind exosome‐based delivery as a promising strategy.

Strong experimental evidence supporting the feasibility of exosome‐mediated PROTAC delivery has been provided by recent studies [11, 16], demonstrating the successful use of camel milk‐derived exosomes (CMEs) to deliver ARV‐825, a bromodomain‐containing protein 4 (BRD4)‐targeting PROTAC, resulting in significantly improved bioavailability and therapeutic efficacy. The CME‐based formulation exhibited high encapsulation efficiency (42.75%), nanoscale particle size (136.8 nm), and favourable zeta potential (−32.75 mV), contributing to enhanced stability and sustained drug release. Notably, this delivery system achieved a 3.2‐fold increase in permeability, reduced IC_50_ values in cancer cell lines, and a five‐fold increase in oral bioavailability in vivo, underscoring the transformative potential of exosome‐based PROTAC delivery [15, 16, 22, 23]. Beyond natural exosomes, engineered exosomes further strengthen the rationale for this delivery approach. Advances in exosome engineering have enabled improved control over heterogeneity, scalability, and payload loading efficiency. The use of well‐characterized cell lines for exosome production enhances batch consistency and manufacturing reproducibility, addressing key challenges associated with clinical translation [24]. Additionally, engineered exosomes can be loaded with diverse therapeutic payloads, including small molecules, siRNAs, miRNAs, mRNAs, CRISPR/Cas9 components, and proteins, demonstrating their versatility as multifunctional delivery platforms [15, 16, 25, 26]. Although encapsulation efficiency may vary depending on cargo size, PROTACs fall within the range of molecules that can be effectively loaded using existing techniques such as electroporation and microfluidic systems. Despite these advantages, challenges remain, particularly with respect to large‐scale exosome production, purification, and standardisation. Achieving high purity and sufficient yield continues to be a technical hurdle, and further optimisation is required before widespread clinical adoption is feasible. Nevertheless, the cumulative evidence strongly supports exosome‐based delivery as a rational and promising strategy to overcome the intrinsic limitations of conventional PROTAC therapeutics (Figure 5).

The protagonist rationale for exosome‐based PROTAC delivery is grounded in the unique biological properties of exosomes, including their stability, biocompatibility, targeting potential, and ability to cross biological barriers. By enhancing PROTAC stability, improving intracellular delivery, reducing off‐target toxicity, and enabling tissue‐specific targeting, exosomes offer a biologically integrated solution to the major challenges limiting PROTAC translation. Continued refinement of exosome engineering and manufacturing strategies is expected to further advance this approach, paving the way for next‐generation targeted protein degradation therapies.

## 4 Engineered Exosomes for PROTAC Delivery

While naturally secreted exosomes already possess several advantages as drug delivery vehicles, their native heterogeneity, variable cargo composition, and limited loading efficiency necessitate further modification to meet the stringent requirements of therapeutic applications. Engineered exosomes have therefore emerged as an advanced and rational evolution of exosome‐based delivery systems, offering enhanced control over cargo loading, targeting specificity, reproducibility, and scalability. In the context of PROTAC therapeutics, exosome engineering is particularly valuable, as it addresses the intrinsic physicochemical and pharmacokinetic limitations of PROTAC molecules while preserving the biological advantages of exosomes [15, 16, 25, 26]. One of the primary motivations for engineering exosomes is to overcome heterogeneity associated with naturally derived extracellular vesicles. Exosomes isolated from biological fluids or primary cells often display substantial variability in size, surface protein composition, and cargo content, which can affect therapeutic consistency and reproducibility. The use of established and well‐characterized cell lines for exosome production has been shown to significantly improve homogeneity and batch‐to‐batch consistency, thereby facilitating downstream manufacturing and regulatory compliance [24]. Engineered exosomes produced from stable cell lines allow better control over chemistry, manufacturing, and quality attributes, which is essential for the clinical translation of complex therapeutics such as PROTACs. Cargo loading represents another critical aspect of exosome engineering. Native exosomes naturally carry proteins, lipids, and nucleic acids; however, the passive incorporation of exogenous therapeutic molecules such as PROTACs is often inefficient. To enhance loading efficiency, several active loading strategies have been developed, including electroporation, sonication, extrusion, freeze thaw cycling, and microfluidic‐based techniques [15, 16, 22, 23, 24, 27, 28, 29]. Among these, electroporation has been widely adopted due to its ability to transiently permeabilize the exosomal membrane, allowing encapsulation of small molecules without permanently compromising vesicle integrity. More recently, microfluidic droplet‐based electroporation systems have demonstrated superior performance by enabling continuous‐flow, low‐voltage loading that preserves exosome structure while achieving high encapsulation efficiency [15, 16]. In the specific case of PROTAC delivery, these advanced loading techniques are particularly relevant. PROTACs, despite being larger than conventional small molecules, fall within a size range that permits effective encapsulation within exosomes when optimized loading strategies are employed. Studies have shown that engineered exosomes can encapsulate PROTACs with high efficiency while maintaining favourable physicochemical properties such as nanoscale size and negative zeta potential, which are critical for circulation stability and cellular uptake [16]. Importantly, encapsulation within engineered exosomes protects PROTACs from enzymatic degradation and improves their pharmacokinetic profile, leading to sustained drug release and enhanced intracellular delivery.

Surface engineering of exosomes further strengthens their utility as PROTAC delivery vehicles by enabling active targeting of specific tissues or cell types. Exosomal membranes naturally express a variety of surface proteins, including tetraspanins, integrins, and adhesion molecules, which influence their biodistribution and cellular uptake (Van Niel et al., 2018). Through genetic or chemical modification, these surface features can be tailored to enhance targeting specificity. For example, the display of targeting ligands, antibodies, or peptides on exosomal surfaces has been shown to promote selective binding to cancer cells, infected cells, or inflamed tissues [27, 28] Such modifications are particularly advantageous for PROTAC therapeutics, as they reduce nonspecific tissue distribution and minimize off‐target protein degradation.

**FIGURE 6:**
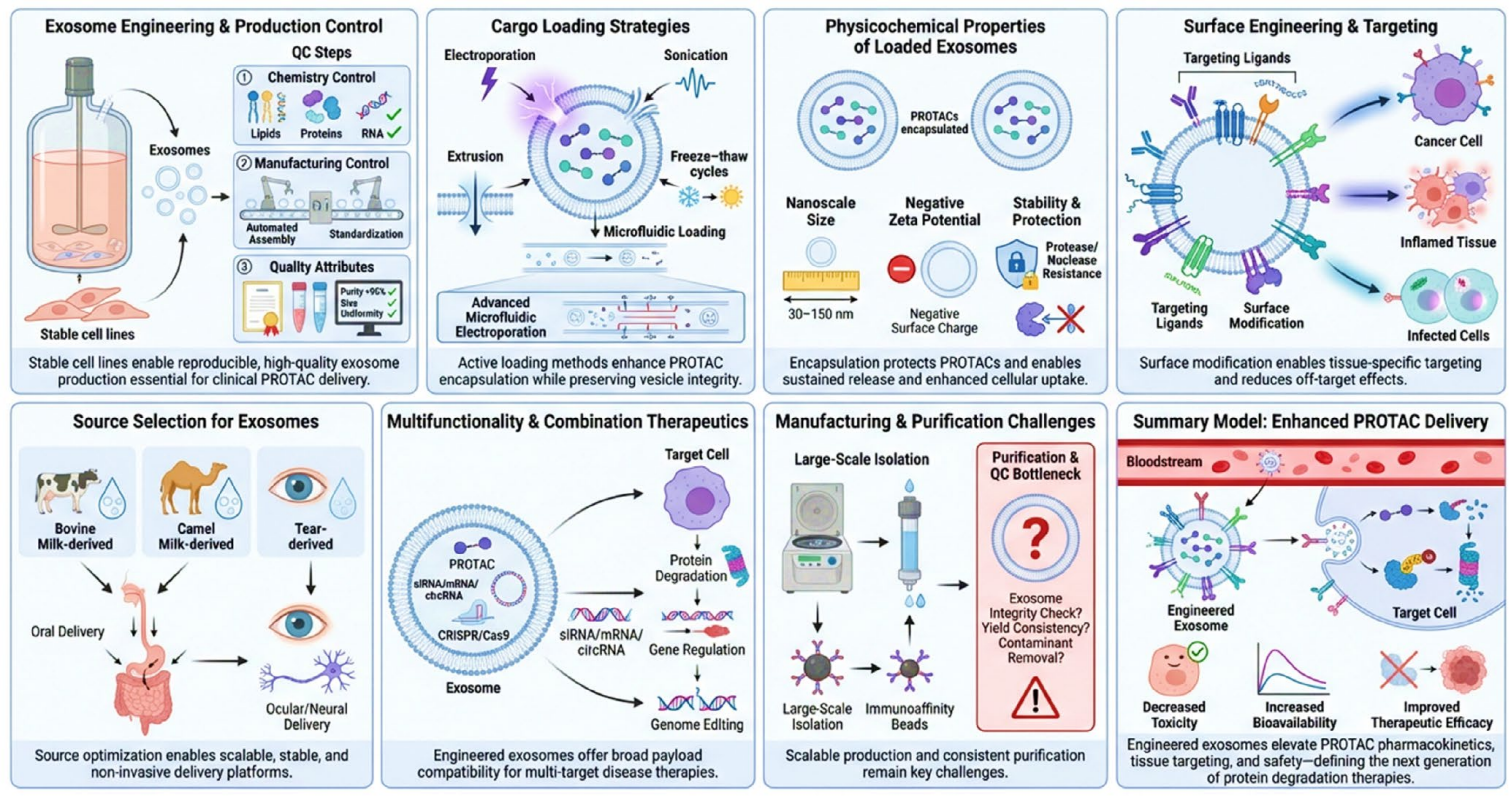
Various surface engineering strategies for exosome surface modification.

**FIGURE 7:**
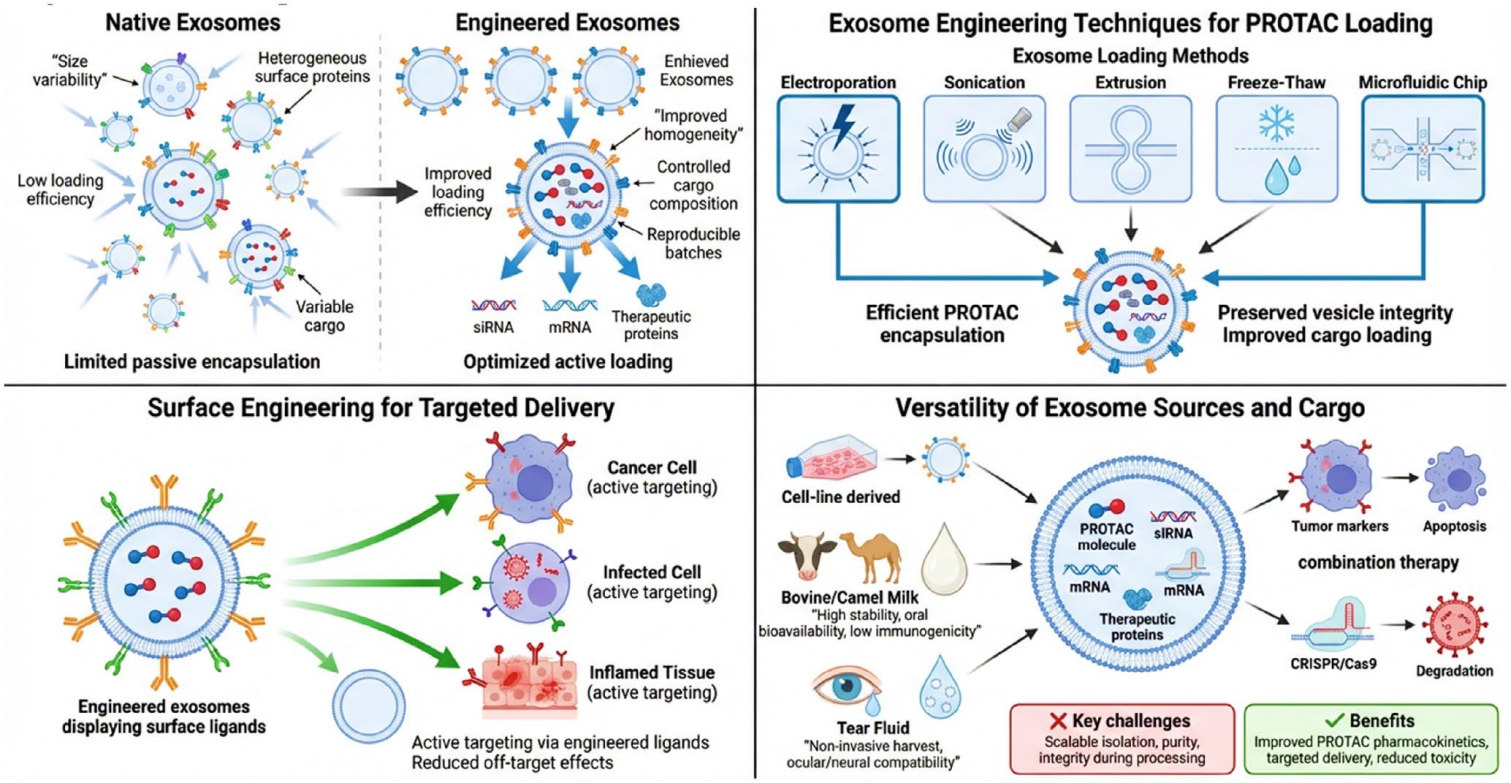
Various exosome engineering strategies for enhanced drug delivery and minimizing off‐target effects.

Engineered exosomes (Figures 6 and 7) also offer exceptional versatility in terms of payload compatibility. Beyond small molecules like PROTACs, engineered exosomes have been successfully loaded with siRNAs, miRNAs, mRNAs, circular RNAs, CRISPR/Cas9 components, and therapeutic proteins, highlighting their adaptability as multifunctional delivery platforms [15, 16, 25, 26]. This versatility opens the possibility of combination strategies in which PROTACs are co‐delivered with nucleic acid therapeutics to achieve synergistic effects, such as simultaneous protein degradation and gene regulation. Such approaches may be particularly useful in complex diseases like cancer and viral infections, where multi‐target modulation is often required. The choice of exosome source plays a crucial role in determining delivery efficiency and therapeutic outcome. Milk‐derived exosomes, including those isolated from bovine and camel milk, have attracted significant attention due to their scalability, stability, and oral bioavailability [16, 29]. Camel milk‐derived exosomes (CMEs) have demonstrated robust stability, low immunogenicity, and efficient drug loading capacity [11, 16]. Reported that CMEs loaded with the BRD4‐targeting PROTAC ARV‐825 exhibited enhanced permeability, prolonged circulation, and significantly improved bioavailability compared to free drug. These findings underscore the value of engineered milk‐derived exosomes as practical and translatable platforms for PROTAC delivery.

Tear‐derived exosomes represent another innovative and non‐invasive source for engineered exosome‐based delivery. Exosomes present in tear fluid have been shown to carry biologically active proteins and RNAs and can be harvested without invasive procedures [14, 17]. Their natural compatibility with ocular and neural tissues makes them particularly attractive for delivering PROTACs targeting viral infections, ocular diseases, and neurodegenerative disorders. Engineering tear exosomes to express specific targeting ligands further enhances their therapeutic potential while minimising immune activation, which is especially important in immunocompromised patient populations.

Despite the significant promise of engineered exosomes, challenges remain that must be addressed to fully realize their clinical potential. Large‐scale production of high‐purity exosomes with consistent quality remains technically demanding. Isolation and purification methods, such as ultracentrifugation, size exclusion chromatography, and immunoaffinity capture, vary in efficiency and scalability, and no single method has yet emerged as universally optimal [19]. Moreover, maintaining exosome integrity during loading and storage is critical to preserving their biological function and delivery capability.

Engineered exosomes signify a sophisticated and biologically integrated delivery platform that directly addresses many of the limitations associated with conventional PROTAC therapeutics. Through controlled cargo loading, surface functionalization, and source optimization, engineered exosomes enhance PROTAC stability, improve intracellular delivery, enable tissue‐specific targeting, and reduce systemic toxicity. Continued advances in exosome engineering, manufacturing, and quality control are expected to further strengthen this approach, positioning engineered exosomes as a cornerstone technology for next‐generation targeted protein degradation therapies.

## 5 Therapeutic Applications of Exosome‐Based PROTAC Delivery

The integration of exosome biology with PROTAC technology represents an emerging class of biologically vectored degraders with broad therapeutic potential. PROTACs offer a fundamentally distinct pharmacological strategy by inducing catalytic degradation of target proteins through recruitment of E3 ubiquitin ligases, rather than relying on continuous target occupancy. However, the clinical translation of many PROTACs has been limited by poor oral bioavailability, rapid systemic clearance, restricted tissue penetration, and off‐target toxicity. Exosomes, as naturally occurring nanoscale extracellular vesicles, provide a biologically evolved lipid bilayer platform capable of encapsulating bulky or lipophilic PROTACs, protecting them from premature degradation, and facilitating targeted cellular uptake through surface adhesion molecules, integrins, and tetraspanins. By combining these properties, exosome–PROTAC systems aim to address key pharmacokinetic and pharmacodynamic barriers that have constrained PROTACs largely to preclinical development.

### 5.1 In Targeting Viral Infections

RNA viruses such as SARS‐CoV, MERS‐CoV, influenza viruses, Ebola virus, and human immunodeficiency virus (HIV) depend on a limited set of viral proteins and host cofactors that are essential for replication, immune evasion, and persistence. These proteins are particularly well suited for degradation‐based therapeutic strategies. Unlike conventional antiviral inhibitors that rely on reversible binding to enzymatic active sites, PROTACs enable catalytic, sub‐stoichiometric elimination of target proteins. This distinction is important in viral diseases, where resistance often emerges through single amino‐acid substitutions that reduce inhibitor binding but preserve enzymatic function.

In Ebola virus infection, the VP24 protein plays a critical role in antagonizing host innate immunity by binding karyopherin‐α (KPNA) and preventing STAT1 nuclear translocation, thereby suppressing type I interferon signalling. PROTACs designed to degrade VP24, using warheads derived from VP24‐host interface inhibitors linked to CRBN or VHL ligands, could restore JAK–STAT signalling and re‐enable antiviral defence mechanisms, particularly during early systemic infection.

In SARS‐CoV‐2, the main protease (M^pro^, also known as 3CL^pro^) is indispensable for processing viral polyproteins into functional non‐structural proteins required for replication complex formation. Numerous covalent and non‐covalent M^pro^ inhibitors have been developed, many of which could be repurposed as PROTAC warheads. By driving proteasomal degradation rather than reversible inhibition, M^pro^‐targeting PROTACs may retain efficacy even against viral variants with reduced inhibitor affinity, since restoration of protease activity would require de novo protein synthesis to outpace degradation. Host factors involved in viral entry and replication, such as ACE2, TMPRSS2, cyclophilins, and viral accessory proteins that suppress innate immunity (including SARS‐CoV‐2 ORF6 and ORF9b), are also conceptually attractive PROTAC targets. However, degradation of host proteins raises important safety concerns, particularly with systemic exposure. In this context, exosome‐based delivery offers a critical advantage by enabling localized or cell‐type‐restricted delivery, thereby reducing unintended protein loss in healthy tissues.

#### 5.1.1 Exosome‐Mediated Antiviral PROTAC Delivery

Exosomes can be engineered from permissive or disease‐relevant cell types, such as airway epithelial cells, macrophages, or CD4^+^ T cells, to enhance homing to infected tissue compartments. Their lipid bilayer structure protects labile or highly lipophilic PROTACs from plasma hydrolysis and hepatic first‐pass metabolism, both of which often limit systemic exposure of free drug. In addition, exosomes are efficiently internalized by recipient cells through endocytosis or membrane fusion mediated by native ligands, including integrins, phosphatidylserine, and tetraspanins, resulting in higher intracellular drug concentrations than those achieved with unencapsulated PROTACs. An additional advantage of exosome‐based antiviral delivery is the potential for co‐loading immunomodulatory cargo. For example, antiviral PROTACs could be combined with nucleic acid payloads that activate innate immune pathways, such as STING agonists or interferon‐mimetic RNAs, allowing simultaneous depletion of viral proteins and restoration of suppressed antiviral signalling [30, 31, 32, 33, 34], and subsequent mechanistic studies have shown that exosome‐loaded antiviral PROTACs reduce viral protein abundance, restore interferon‐stimulated gene expression, and decrease viral titters in infected cell models, although these findings remain largely preclinical.

**FIGURE 8:**
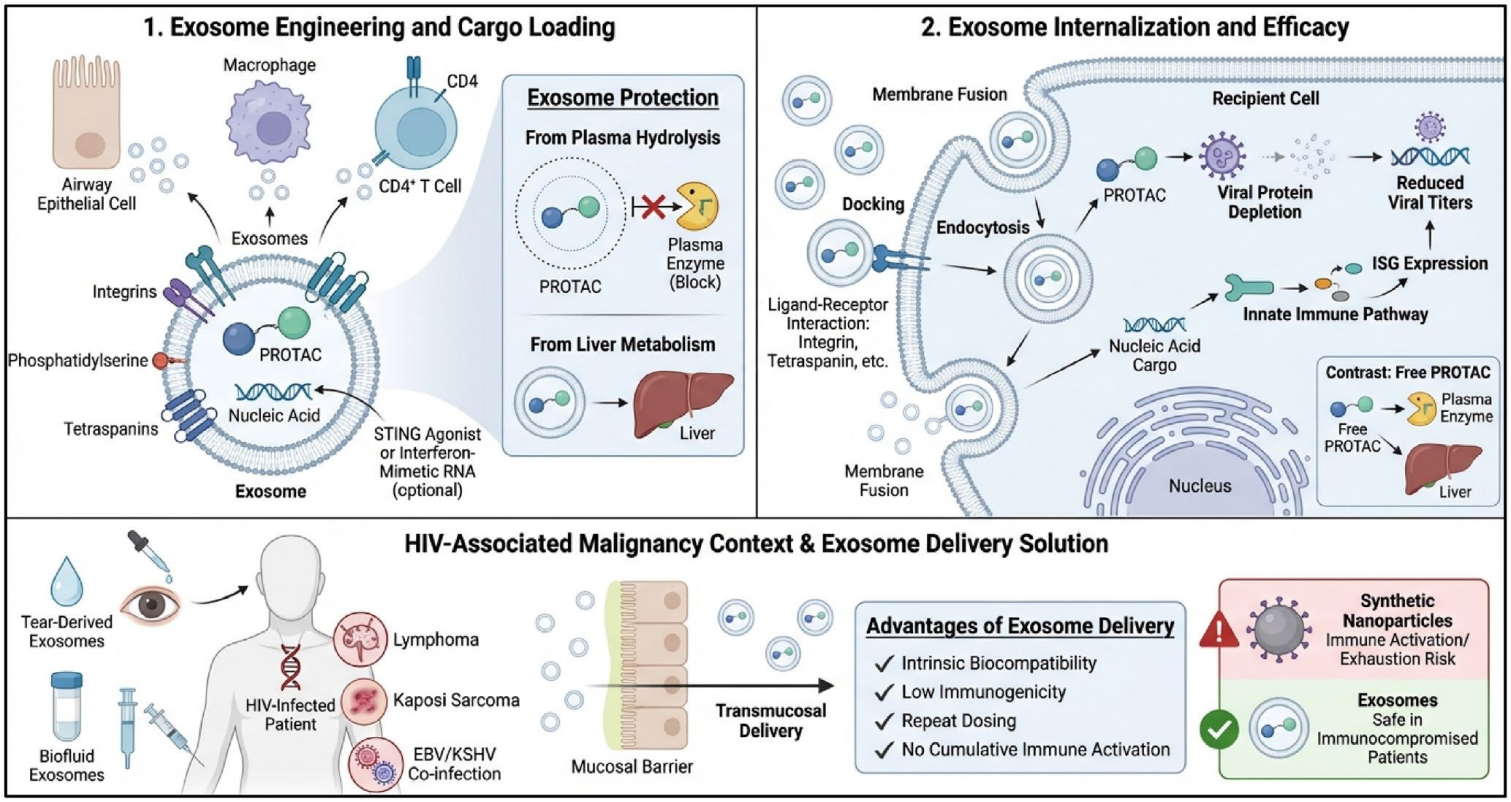
Engineered exosome for targeted delivery of PROTACs in HIV and subsequent viral infections.

Chronic HIV infection is characterized by persistent viral reservoirs and long‐term immune dysfunction, which significantly increases the risk of virus‐associated malignancies, including lymphomas, Kaposi sarcoma, and cancers driven by co‐infection with Epstein–Barr virus or Kaposi sarcoma associated herpesvirus. PROTACs targeting viral oncoproteins or host factors that sustain oncogenic signalling represent a promising strategy in this setting, but delivery challenges are particularly acute in immunocompromised patients. Tear‐derived exosomes and other non‐invasively harvested biofluid exosomes have been proposed as carriers for PROTACs targeting viral oncogenes such as LMP1 or KSHV LANA. These exosomes exhibit intrinsic biocompatibility, low immunogenicity, and the ability to cross mucosal barriers, making them suitable for repeated administration without provoking cumulative immune activation. Such properties are especially important in HIV‐positive individuals, where synthetic nanoparticle systems may exacerbate inflammation or immune exhaustion (Figure 8) (Table 1).

**TABLE 1:** Exosome‐based delivery strategies for PROTAC‐mediated targeting of viral infections [21].

Virus	Viral protein	Function of viral protein	Potential effect of PROTAC/mechanism	Potential exosomes for PROTAC delivery
Ebola virus	VP24	Inhibits interferon signalling	Restoration of antiviral response of host	Exosomes derived from immune cells
SARS‐CoV‐2	Main protease	Required for viral replication	Inhibition of viral replication	Exosomes derived from lung epithelial cells
MERS‐CoV	3CL protease	Involved in processing viral polyproteins	Inhibition of viral polyprotein processing	Exosomes derived from lung epithelial cells
H1N1 influenza	Neuraminidase	Involved in release of progeny viruses	Inhibition of viral spread	Exosomes derived from lung epithelial cells
H5N1 avian influeza	Hemagglutinin	Involved in viral entry	Inhibition of viral entry	Exosomes derived from lung epithelial cells
H7N9 avian influenza	Polymerase acidic protein (PA)	Involved in viral replication	Inhibition of viral replication	Exosomes derived from lung epithelial cells
Zika virus	NS5	Involved in viral replication and immune evasion	Inhibition of viral replication and restoration of immune response	Exosomes derived from neural cells
Nipah virus	W protein	Involved in immune evasion	Restoration of immune response	Exosomes derived from endothelial cells
Hantavirus	N protein	Involved in viral replication	Inhibition of viral replication	Exosomes derived from endothelial cells
Dengue virus	NS3	Involved in viral replication	Inhibition of viral replication	Exosomes derived from immune cells

### 5.2 In Targeting Cancer Therapy

Cancer remains the most advanced application area for PROTACs, with several small‐molecule degraders, including ARV‐110 and ARV‐471, already in clinical trials. Exosome‐based delivery systems are now being explored to further improve the therapeutic index of these agents by enhancing tumour accumulation and reducing systemic toxicity. Exosomes can exploit both passive targeting mechanisms, such as enhanced permeability and retention in tumours, and active targeting mediated by tumour‐tropic integrins, adhesion molecules, and chemokine receptors. A notable proof‐of‐concept is the use of camel milk‐derived exosomes (CMEs) to deliver ARV‐825, a BRD4‐targeting PROTAC [11, 35]. Demonstrated that CME‐encapsulated ARV‐825 exhibited substantially improved pharmacokinetic and pharmacodynamic properties compared with free drug. The formulation showed high entrapment efficiency, nanoscale particle size, and a negative zeta potential conducive to colloidal stability. In vitro, CME‐ARV‐825 displayed enhanced permeability and lower IC50 values in resistant cancer cell lines, while in vivo oral administration resulted in significantly increased systemic exposure and bioavailability. These findings provide strong evidence that milk‐derived exosomes can convert a PROTAC traditionally requiring parenteral administration into an orally viable therapeutic. Beyond PROTACs, multiple studies have demonstrated the utility of exosomes as carriers for classical chemotherapeutics, supporting their use in combination strategies. For example, cisplatin‐loaded umbilical cord macrophage‐derived exosomes exhibit enhanced uptake and cytotoxicity in ovarian cancer cells, while bovine milk exosomes carrying phytochemicals such as withaferin A suppress tumour growth in lung cancer xenografts with reduced systemic toxicity. These platforms could be adapted to co‐deliver a PROTAC and a cytotoxic agent, linking targeted protein degradation with replication stress or DNA damage within a single delivery system.

**FIGURE 9:**
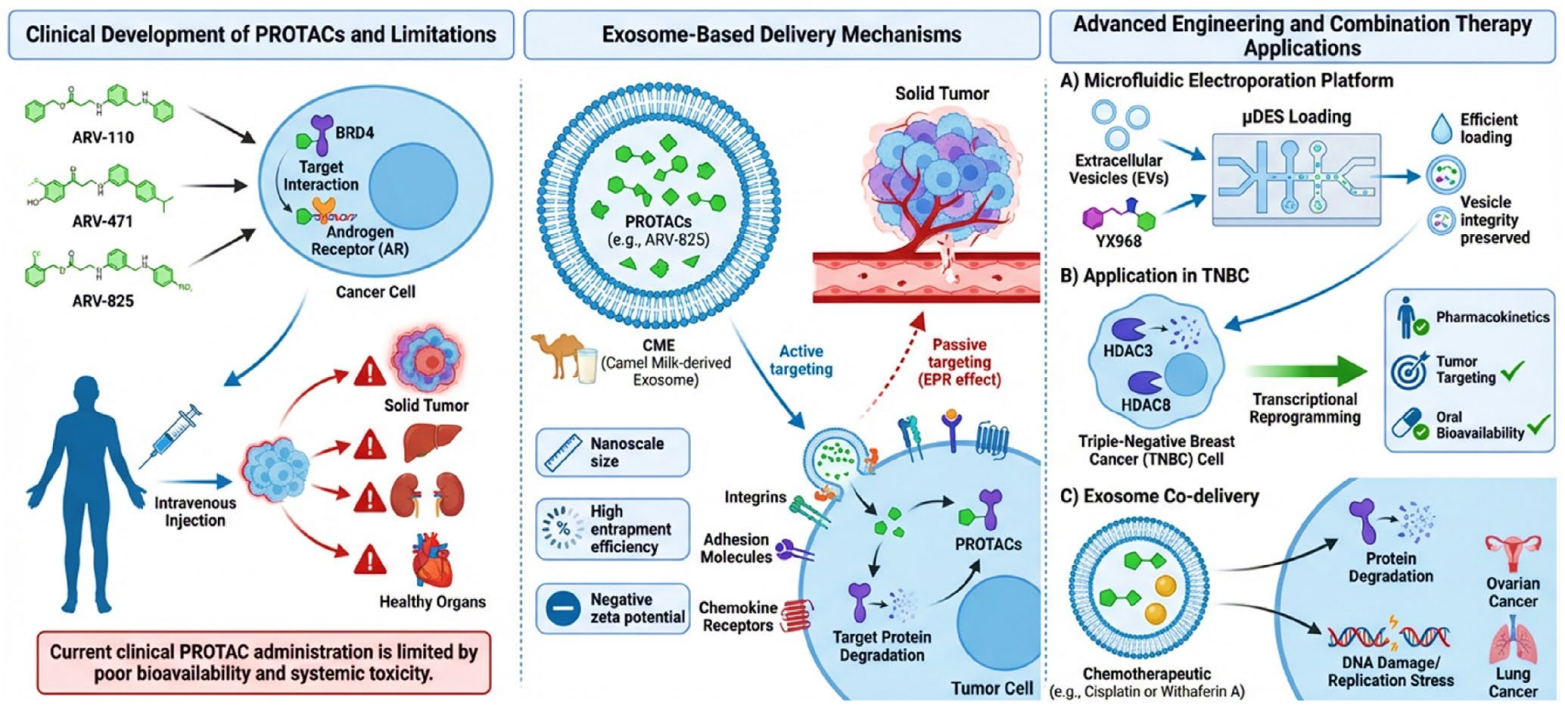
Exosome mediated delivery of PROTACs in various cancer theragnostic.

Triple‐negative breast cancer (TNBC) is characterized by aggressive behaviour, early metastasis, and limited therapeutic options. Class I histone deacetylases, particularly HDAC2, HDAC3, and HDAC8, are frequently overexpressed in TNBC and contribute to proliferation, stemness, and immune evasion. While HDAC inhibitors have shown limited success due to off‐target toxicity, HDAC‐targeting PROTACs achieve more selective degradation and deeper transcriptional reprogramming but remain limited by poor pharmacokinetics and cellular uptake. To address these challenges, a microfluidic droplet‐based electroporation (μDES) platform was developed to load HDAC‐targeting PROTAC YX968 into extracellular vesicles. This approach achieved higher loading efficiency and preserved vesicle integrity compared with conventional methods. In TNBC cell models, EV‐delivered YX968 induced rapid and sustained degradation of HDAC3 and HDAC8 at doses where free PROTAC was minimally effective. In xenograft models, EV‐encapsulated YX968 produced greater target depletion and tumour growth inhibition than equivalent doses of free drug, demonstrating that exosome‐enabled delivery can substantially enhance in vivo efficacy of PROTACs in aggressive solid tumours (Table 2) (Figure 9).

**TABLE 2:** Exosome‐mediated therapeutic strategies and biomarkers in Triple‐Negative Breast Cancer.

Targets	Application	Source	Objective	References
Circulating exosomal miRNAs	TNBC	Sera of TNBC patients	As biomarkers to predict treatment efficacy for TNBC as diagnostic tools	[35]
Exosomal miRNAs may modulate chemoresistance	TNBC	Sera of TNBC patients	Biomarkers of response to pembrolizumab in patients with TNBC	[36]
A15‐ Exo	TNBC	Human macrophages	A15‐ Exo to co‐deliver miRNA and chemotherapeutics via biomimicry	[34]
Proteomic profiling of exosomes	TNBC	Sera of TNBC patients	Circulating exosomal microRNA profiling was established for potential biomarkers and therapeutically targets identification	[37]
Exosomal miRNA profiles	TNBC	Sera of TNBC patients undergoing neoadjuvant chemotherapy	Establishing biomarkers	[38]
Survivin	Breast cancer	Sera of patients with breast cancer	Having early diagnostic value in cancer	[39]

### 5.3 Neurodegenerative Diseases

Neurodegenerative disorders, including Alzheimer's disease (AD), Parkinson's disease (PD), amyotrophic lateral sclerosis (ALS), and frontotemporal dementia, are unified by the progressive loss of neuronal function driven by the accumulation of misfolded or aggregation‐prone proteins such as amyloid‐β, hyperphosphorylated tau, α‐synuclein, and TDP‐43. These pathogenic proteins disrupt cellular proteostasis, impair synaptic function, and ultimately lead to neuronal death. From a mechanistic standpoint, such diseases are well suited to targeted protein degradation strategies, as they involve aberrant protein species that are poorly cleared by endogenous chaperone‐mediated or autophagic pathways. However, the effective translation of degradation‐based therapeutics to the central nervous system (CNS) remains challenging, primarily due to the restrictive nature of the BBB and the presence of active efflux transporters that limit intracellular drug accumulation.

#### 5.3.1 Exosomes as CNS Delivery Shuttle

Exosomes have emerged as promising shuttles for CNS drug delivery because of their intrinsic ability to traverse the BBB. Multiple studies indicate that exosomes cross the BBB through receptor‐mediated transcytosis and interactions with endothelial cells, microglia, and neurons. Preclinical investigations using exosomes loaded with small molecules, siRNAs, and miRNAs have demonstrated robust CNS penetration and biologically meaningful effects. In AD models, exosomes engineered to display neuron‐specific or peptide‐based targeting motifs reduce amyloid‐β deposition, attenuate tau phosphorylation, suppress microglial activation, and improve memory performance. Similarly, in PD models, exosomal delivery of α‐synuclein‐targeting siRNAs or small molecules has been shown to reduce α‐synuclein aggregation, preserve dopaminergic neurons, and improve motor function. Although direct applications of exosome‐mediated PROTAC delivery in neurodegenerative disease remain largely conceptual, the mechanistic alignment is strong. Misfolded proteins that resist normal degradation pathways represent ideal candidates for PROTAC‐based elimination. The ability of exosomes to deliver cargo efficiently into neurons and glial cells suggests that they could overcome one of the principal limitations of CNS‐directed PROTACs, namely insufficient intracellular exposure.

#### 5.3.2 Emerging CNS PROTAC Targets and Translational Considerations

**FIGURE 10:**
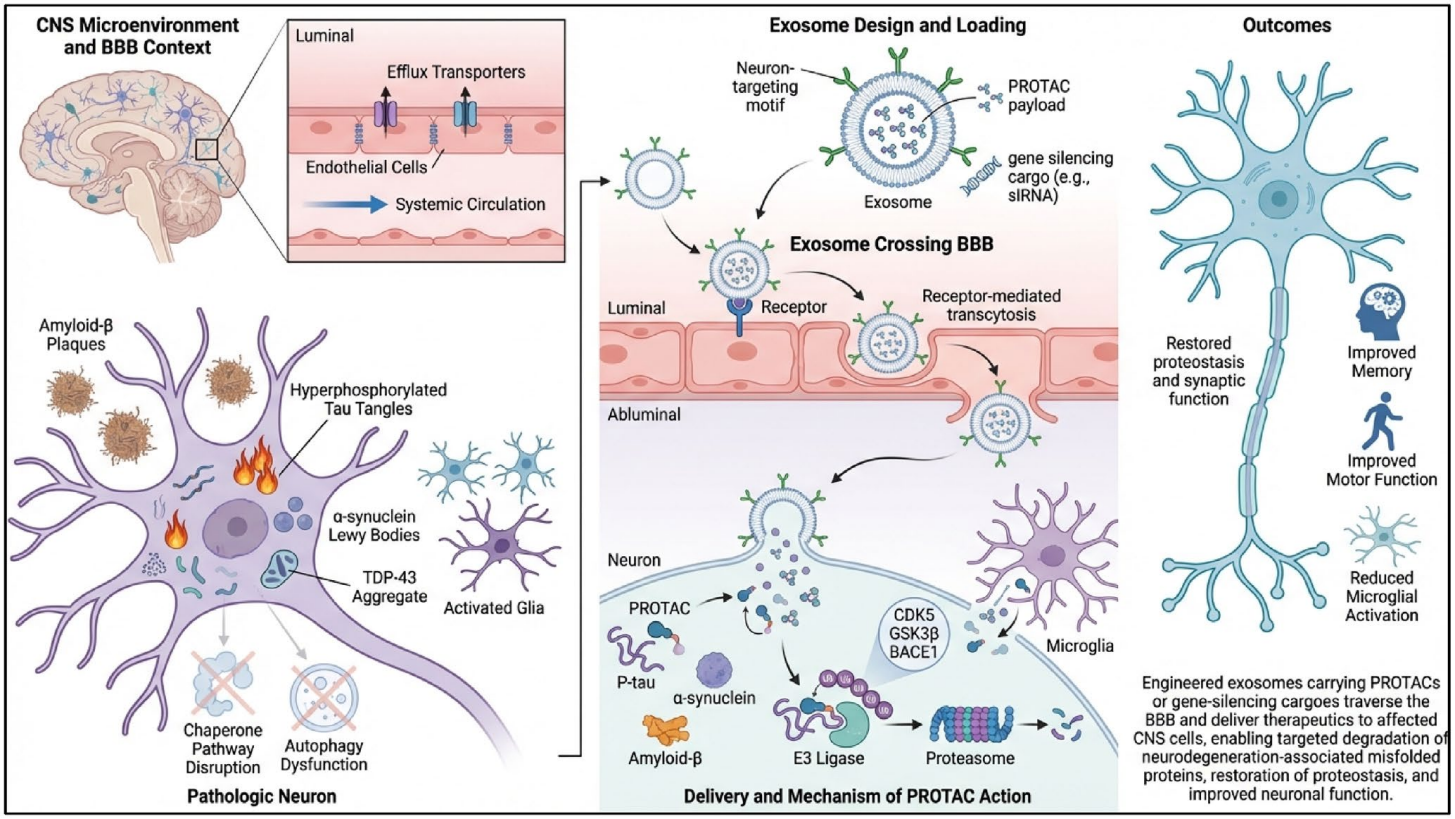
Exosome mediated delivery of PROTACs in various neurodegenerative disease theragnostic.

Ongoing efforts in PROTAC design are beginning to focus on CNS‐relevant targets that are directly implicated in neurodegenerative pathology. These include specific tau isoforms and regulatory enzymes involved in tau phosphorylation, such as cyclin‐dependent kinase 5 (CDK5), glycogen synthase kinase‐3β (GSK3β), and regulators of protein phosphatase 2A (PP2A). In Parkinson's disease, α‐synuclein itself, as well as chaperones and ubiquitin ligases involved in Lewy body formation, are being explored as potential degradation targets. Enzymes involved in amyloidogenic processing, including β‐secretase (BACE1) and components of the γ‐secretase complex, are also attractive candidates, as catalytic degradation may provide more durable disease modification than transient enzymatic inhibition. Importantly, parallel clinical experience with exosome‐based CNS therapies supports the feasibility of this approach. MSC derived exosomes are currently being investigated in neuroinflammatory conditions and stroke, with early studies reporting favourable safety and tolerability profiles. These findings suggest that exosome PROTAC constructs, once optimized, may be acceptable from immunological and toxicological perspectives in the CNS setting (Figure 10) (Tables 3 and 4).

**TABLE 3:** Exosome‐based therapeutic approaches evaluated in Alzheimer's disease models.

Exosomes sources	Therapeutic cargos	Targets/Administration methods	Models	Outcomes	References
BMSCs	miR‐29b	BACE1, BIM	Aβ‐treated model rats	Reduced deficits in spatial learning and memory in a rat model of AD	[40]
	—	Lateral ventricle administration	APP/PS1 mice	Down‐regulated IL‐1β, IL‐6, TNF‐α and up‐regulated BDNF levels	[41]
	miR‐146a	NF‐κB pathways	APP/PS1 mice	Restored astrocyte function and reduced NF‐κB levels	[42]
	—	Aβ	5XFAD mice	Improved cognitive function and reduced Aβ plaques in the hippocampus	[43]
MSCs	miR‐29	HDAC4	hAPP‐J20 mice	Improved cognitive function and reduced Aβ levels	[44]
	miR‐223	PTEN	Aβ‐treated SH‐SY5Y cells	Reduced apoptosis in neuronal cells by PTEN‐PI3K/Akt	[45]
	RVG	Cortical and hippocampal	APP/PS1 mice	Reduced cognitive impairment and decreased levels of inflammatory factors	[46]
	—	Intranasal route administration	3×Tg mice	Reduced microglial activation and increased dendritic spine density	[47]
HAFSCs	miR‐21	Aβ	APP/PS1 mice	Decreased Aβ levels and activated STAT3 and NF‐κB levels	[48]
	—	—	LPS‐BV2 microglia	Suppressed elevation of oxidative stress and cell apoptosis in neurons	[49]
ADMSCs	Circ‐Epc1	BV2 microglia	APP/PS1 mice	Improved cognitive function and reduced neuronal damage	[50]
	miR‐22	GSDMD	APP/PS1 mice	Decreased expression of inflammatory cytokines and GSDMD	[51]
NSCs	—	Aβ	Aβ‐induced C57BL/6 mice	Alleviated memory deficits	[52]
	miRNA	Aβ	5XFAD mice	Reduced Aβ deposition and cognitive deficits	[53]
	—	Aβ	APP/PS1 mice	Reduced cognitive deficits	[54]
Bioengineered microglia‐targeting exosomes	Gemfibrozil	Aβ	Aβ‐treated model rats	Reduced Aβ level and improved learning and memory abilities	[55]

**TABLE 4:** Exosome‐based delivery systems investigated in Parkinson's Disease models.

Exosome sources	Therapeutic cargos	Targets	Models	Outcomes	References
HUMSCs	—	—	6‐OHDA‐induced PD rats; SHSY5Y cell	Alleviated behavioural deficits and decreased DN loss	[56]
BMSC	—	Striata	PD rats	Reduced levels of IL‐6, IL‐1β, TNF‐α, and ROS; recovered rotation behaviour and climbing speed in rats	[41]
	ASO	α‐Syn	α‐syn A53T mice	Reduced α‐syn expression	[57]
	Gli1	Sp1 signalling pathway	MPTP‐induced PD mice	Reduced neuronal damage and inflammatory responses	[58]
	—	α‐Syn	Two *C. elegans* models	Reduced expression level of α‐syn	[59]
ADMSC	miR‐188‐3p	CDK5, NLRP3	MPTP‐induced PD mice	Inhibited autophagy and clocking in the PD model and increased proliferation of M9ND cells	[60]
MSCs	—	SMAD3, p38 MAPK	MPTP‐injected mice	Promoted angiogenesis	[61]
	—	—	6‐OHDA‐injected rats	Reduced motor deficits and prevented against TH injury	[62]
	RVG	α‐Syn	PD mice	Significantly improved motor behaviour	[63]
NSCs	—	—	6‐OHDA‐induced mice	Decreased ROS levels; reduced DN loss	[64]
Dendritic cell	Redox catalase	ROS	PD mice	Reduced ROS levels, achieving neuroprotective effects	[65]
	shRNA	α‐Syn	Syn PFFs‐injected mice	Reduced α‐syn aggregation and neuronal death	[66]
	α‐syn‐siRNA	α‐Syn	S129D α‐syn transgenic mice	Significantly reduced protein aggregates within neurons	[67]
Blood	Dopamine	Dopamine receptors	6‐OHDA‐injected mice	Reduced DN degeneration	[68]
Exotic equipment	Catalase mRNA	ROS	6‐OHDA‐injected rats	Inhibited neuroinflammation	[69]
Serum	miR‐137	OXR1	PD mice	Reduced oxidative stress damage of PD	[70]

### 5.4 Inflammatory and Autoimmune Diseases

**FIGURE 11:**
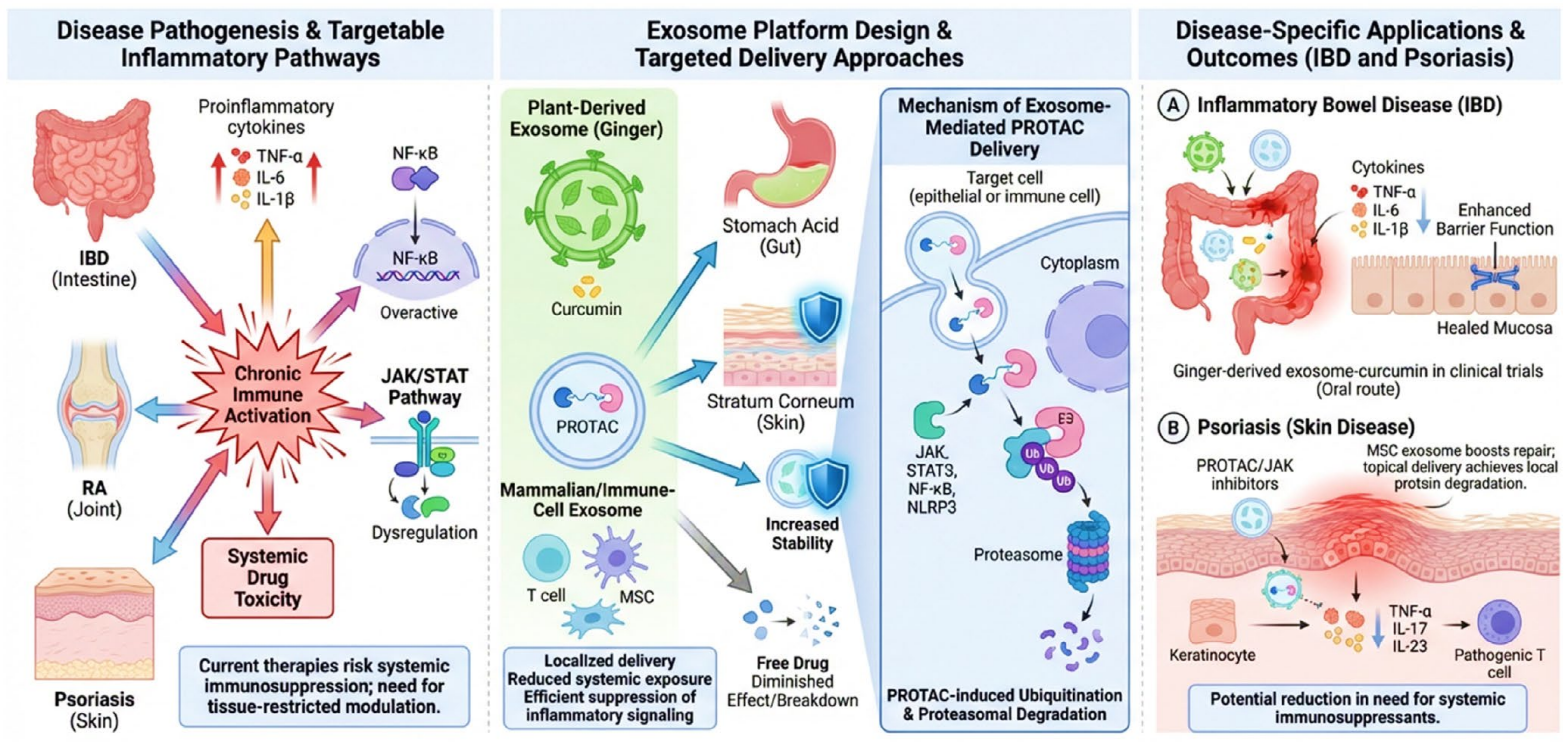
Exosome mediated delivery of PROTACs in various inflammatory and autoimmune diseases theragnostic.

Chronic inflammatory and autoimmune disorders, including inflammatory bowel disease (IBD), psoriasis, rheumatoid arthritis, and systemic inflammatory syndromes, are driven by sustained activation of cytokine networks, transcription factors such as NF‐κB and STATs, and upstream kinases that perpetuate immune dysregulation. Many of these signalling components are amenable to selective degradation, making PROTAC‐based approaches conceptually attractive. However, systemic inhibition or degradation of inflammatory mediators often leads to dose‐limiting toxicity and immunosuppression, highlighting the need for localized or tissue‐restricted delivery strategies (Figure 11) (Table 5).

**TABLE 5:** Engineered exosome platforms for immunomodulatory and anti‐inflammatory therapeutic applications [71].

Source	Modification strategy	Cargo	Effects and mechanisms
Bone marrow mesenchymal stromal cells	Surface modification	Photocrosslinked spherical gelatin methacryloyl hydrogel encapsulated cartilage affinity WYRGPL peptide	Strengthen the inhibition of pro‐inflammatory factor IL‐1β
Placental mesenchymal stem cells	Surface modification	Exosomes were encapsulated with N‐(2‐hydroxy) propyl‐3‐trimethyl chitosan ammonium chloride and oxidized konjac glucomannan polysaccharide	Inhibition of MAPK/NF‐κB signalling pathway enhances anti‐inflammatory and tissue repair effects
Macrophages	Surface modification	Modified with both oligolysine and matrix metalloproteinase‐cleavable polyethylene glycol on the membrane	Strengthen the removal of cfDNA and promote macrophage polarization to M2 type
Chondrocyte	Membrane fusion	The chondrocyte affinity peptide was fused with the lysosomal associated membrane glycoprotein 2b protein on the surface of exosomes	Delivery of miR‐140, inhibition of cartilage‐degrading proteases and alleles
Placental mesenchymal stem cells	Membrane fusion	SFBP‐Gluc‐MS2 was constructed by silk fibroin binding peptide, and related SGM‐Exos, miR‐146a‐Exos and SGM‐miR146a‐Exos were isolated	Effectively load miR‐146a to promote chronic wound healing
Exosomes	Cargo loading	Loading OVA antigen	Regulating CD8^+^ T cell immune response
Fibroblasts	Cargo loading	Loading Tripterygium wilfordii	Reduce the expression of pro‐inflammatory factors IL‐6, IL‐18 and IL‐1α; help exosomes escape the phagocytosis of MPS macrophages
B cells	Electroporation	Loading miR‐155	The expression of TNF‐α decreased; effectively prevent the inflammatory induction of LPS
Adipose mesenchymal stem cells	Gene engineering	Overexpression of miR‐132 by lentivirus transduction	Inhibition of NF‐κB p65 and IκB phosphorylation; promote macrophage polarization to M2 phenotype
Mesenchymal stem cells	Gene engineering	Overexpression of miR‐124a	Reduce inflammation score MH7A, RA FLSs cell lines; proliferation‐related expression decreased
Umbilical vein endothelial cells	Gene engineering	Pretreatment of parental cells with TNF‐α	The expression level of miR‐200a‐3p increased through the miR‐200a‐3p/KLF6/VEGFA axis
Umbilical cord mesenchymal stem cells	Gene engineering	Overexpression of miR‐146a‐5p	MiR‐146a‐5p acts on M1 to reduce inflammation; reduce NOTCH1 expression to accelerate M2 macrophage polarization

#### 5.4.1 Exosome‐Based Anti‐Inflammatory Delivery

A growing body of work has established exosomes as effective carriers for anti‐inflammatory agents. Both plant‐ and mammalian‐derived exosomes loaded with compounds such as curcumin or dexamethasone demonstrate enhanced stability in the gastrointestinal tract, increased local drug concentration at inflamed mucosal sites, and more efficient suppression of pro‐inflammatory cytokines including TNF‐α, IL‐6, and IL‐1β in preclinical IBD models. Notably, these effects are achieved with reduced systemic exposure compared with free drug, suggesting an improved therapeutic index. Clinical translation of this concept is already underway. Ginger‐derived exosomes combined with curcumin are currently being evaluated in patients with IBD, with endpoints focusing on safety, tolerability, mucosal inflammation, and symptom scores following oral administration. Together with prior preclinical findings, these studies support the feasibility of using exosomes as platform carriers for degradation‐based anti‐inflammatory therapies.

#### 5.4.2 Immune Cell‐Derived Exosomes in IBD

Exosomes derived from immune cells, including dendritic cells, T cells, and MSCs, exert intrinsic immunomodulatory effects. These vesicles influence cytokine profiles, promote regulatory T‐cell differentiation, suppress pathogenic Th1 and Th17 responses, and enhance epithelial barrier integrity and mucosal healing. They also interact with the gut microbiota and modulate metabolite signalling pathways relevant to intestinal homeostasis. Engineering immune‐cell exosomes to carry PROTACs directed against central inflammatory mediators such as JAK kinases, STAT3, NF‐κB subunits, or NLRP3 inflammasome components could enable localized, proteasome‐dependent suppression of inflammatory signalling. Recently, targeted protein degradation approaches offer a first direct proof of concept regarding the utility of PROTACs as therapeutic agents for the management of inflammatory diseases. STAT3‐targeting PROTACs have successfully abrogated pathological inflammatory signalling pathways by selective degradation of STAT3, therefore diminishing pro‐inflammatory cytokine expression and immune activation downstream. The degradation of IRAK4 and BRD4 by PROTACs also yielded promising anti‐inflammatory activity against preclinical inflammatory disease models via repression of NF‐κB signalling pathways and cytokine generation. Therefore, the feasibility of an integrated approach of exosome‐mediated drug delivery with targeted inflammation PROTAC drug is plausible [71]. Such an approach has the potential to achieve meaningful disease control while minimizing systemic immunosuppression, a major limitation of current biologic therapies.

#### 5.4.3 Psoriasis and Cutaneous Inflammation

Psoriasis and other chronic inflammatory skin diseases represent additional settings where exosome‐based PROTAC delivery may be particularly advantageous. Exosomes loaded with small‐molecule JAK inhibitors, such as tofacitinib, exhibit enhanced penetration into the dermal and epidermal layers compared with conventional formulations. In animal models, this results in stronger local suppression of keratinocyte‐derived cytokines and reduced activation of pathogenic T‐cell populations. MSC‐derived exosomes further contribute intrinsic immunomodulatory and tissue‐repair functions, supporting re‐epithelialization and reduction of inflammatory infiltrates. Adapting these delivery platforms for cutaneous PROTACs targeting JAKs, STAT3, or NF‐κB could enable topical, highly localized protein degradation within psoriatic plaques, potentially reducing the need for systemic therapy.

## 6 Challenges and Future Directions

**FIGURE 12:**
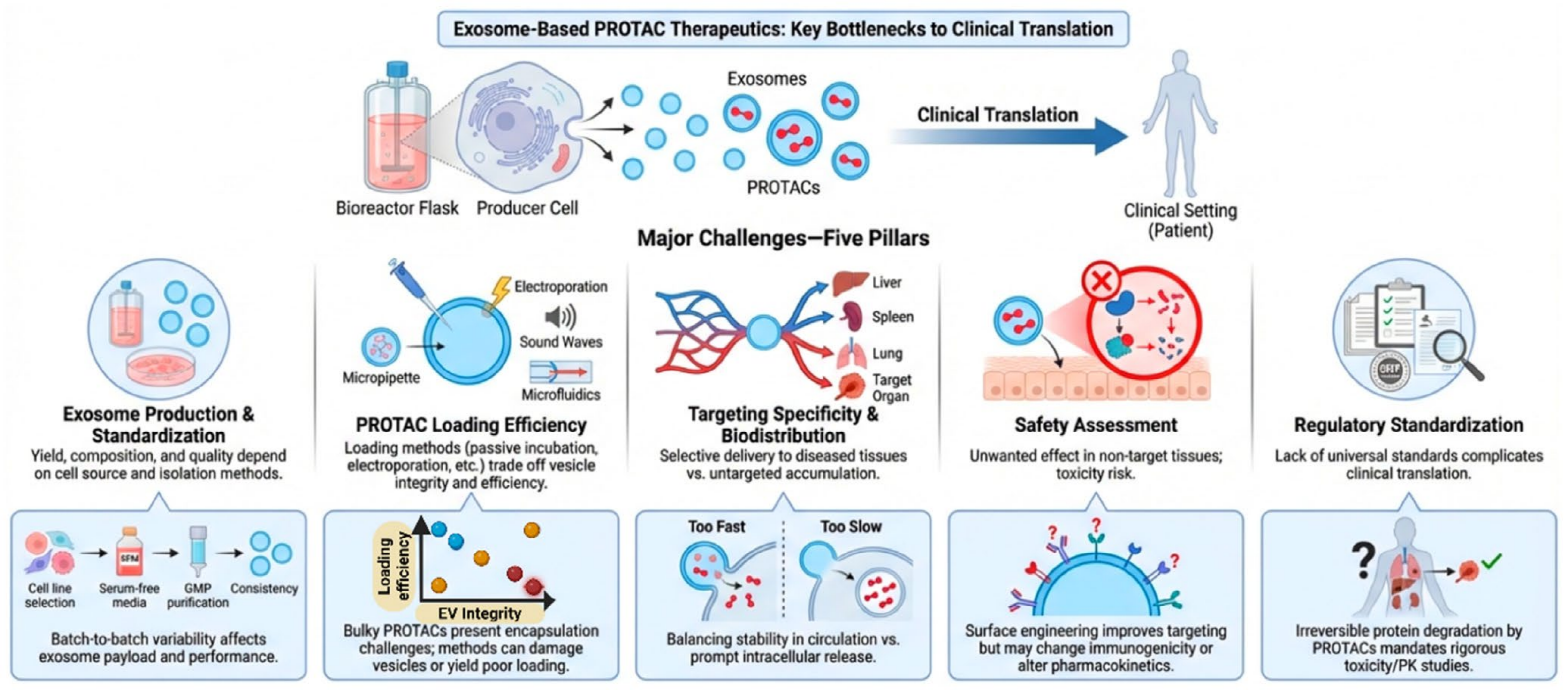
Clinical translation of EV‐PROTACs therapeutics.

Despite the growing body of preclinical evidence supporting exosome‐based delivery of PROTACs, several scientific, technical, and regulatory challenges must be addressed before this platform can be translated into routine clinical use. These challenges span exosome production and characterization, PROTAC loading efficiency, targeting specificity, safety assessment, and regulatory standardization. Addressing these issues in a systematic and evidence‐driven manner will be essential to realize the full therapeutic potential of exosome PROTAC systems. One of the most significant barriers to clinical translation of exosome‐based therapeutics is the lack of scalable, standardized manufacturing processes. Exosomes are biologically derived products, and their yield, composition, and functional properties are highly dependent on the source cell type, culture conditions, and isolation methods [19, 21, 30, 31, 32, 33]. Conventional isolation techniques such as ultracentrifugation, polymer precipitation, and size exclusion chromatography differ widely in purity, recovery, and reproducibility, making cross‐study comparison difficult and complicating regulatory evaluation. For exosome PROTAC systems, this variability is particularly problematic because both the carrier and the payload contribute to therapeutic activity. Batch‐to‐batch inconsistency in exosome surface proteins or lipid composition can alter biodistribution, cellular uptake, and intracellular trafficking of PROTACs. Future efforts must therefore focus on developing robust, scalable bioprocessing pipelines, including well‐defined producer cell lines, serum‐free or chemically defined media, and reproducible purification workflows that meet good manufacturing practice (GMP) standards [8, 33] Efficient and reproducible loading of PROTACs into exosomes remains a technical challenge. While several loading methods have been explored, including passive incubation, electroporation, sonication, and microfluidic approaches, each method presents trade‐offs between loading efficiency, vesicle integrity, and scalability [8]. PROTACs are structurally diverse and often bulky, and their encapsulation efficiency can vary substantially depending on physicochemical properties and loading strategy. In addition to loading efficiency, control over PROTAC release kinetics is critical. Premature release in circulation may negate the benefits of exosomal protection, while excessively slow release may limit intracellular target engagement. Future research should prioritize quantitative studies linking PROTAC loading density, release profiles, and intracellular degradation efficiency. Standardized analytical methods to measure intact PROTAC content, degradation products, and functional activity following delivery are also urgently needed. Although exosomes exhibit intrinsic targeting tendencies based on their cellular origin, their biodistribution following systemic administration is still incompletely understood. Many exosomes accumulate in clearance organs such as the liver, spleen, and lungs, which may reduce delivery efficiency to the intended disease site and increase the risk of off‐target protein degradation [7]. This is particularly relevant for PROTACs, where unintended degradation of target proteins in healthy tissues could result in significant toxicity. Surface engineering strategies, including ligand display or genetic modification of producer cells, offer promising solutions but introduce additional complexity. Each modification must be carefully evaluated for its impact on exosome stability, immunogenicity, and interaction with the mononuclear phagocyte system. Future studies should focus on systematic comparisons of targeting strategies and rigorous in vivo biodistribution analyses using clinically relevant dosing regimens. When combined with PROTACs, which induce irreversible protein degradation, the risk of cumulative or delayed toxicity must be carefully considered. Preclinical safety studies to date have been largely short‐term and focused on acute toxicity. Future investigations must address chronic exposure, immune modulation, and potential effects on endogenous proteostasis networks. This is especially important for applications in neurodegenerative and inflammatory diseases, where long‐term treatment is anticipated. From a regulatory perspective, exosome PROTAC systems present unique challenges because they combine features of biologics, drug delivery systems, and small‐molecule therapeutics. It remains unclear whether such products will be regulated primarily as biological products, advanced therapy medicinal products, or combination drugs. The absence of harmonized regulatory frameworks for extracellular vesicle‐based therapies further complicates clinical development (Figure 12) [33].

Despite these challenges, the future of exosome PROTAC systems is promising. Advances in exosome engineering, including improved control over cargo loading and surface functionalization, are likely to enhance delivery precision and therapeutic index. Parallel progress in PROTAC chemistry, such as the development of ligands for new E3 ligases and optimization of linker design, will further expand the range of druggable targets [1, 4]. Integration of biomarker‐driven patient stratification and real‐time monitoring of target degradation will be essential to identify patient populations most likely to benefit from degradation‐based therapies. Combination strategies, in which exosome‐delivered PROTACs are paired with immunotherapies, chemotherapeutics, or nucleic acid payloads, also warrant careful exploration.

### 6.1 Emerging Agricultural and Environmental Applications of PROTAC Delivery Systems

Beyond clinical medicine, PROTAC concepts are beginning to be explored in agriculture, where selective degradation of plant or pest proteins could provide more sustainable alternatives to traditional agrochemicals. Proposed plant‐directed PROTACs target enzymes such as cytochrome P450s and glutathione S‐transferases, which are implicated in herbicide and pesticide resistance. By degrading these resistance factors, PROTACs could resensitize weeds or pests to lower doses of existing treatments. In principle, exosome‐like vesicles or plant‐derived extracellular vesicles could be engineered for foliar or soil delivery, enabling targeted protein degradation within plant tissues while reducing environmental accumulation associated with broad‐spectrum chemical sprays. Although this field remains at an early conceptual stage, it highlights the modularity of PROTAC design and the versatility of vesicle‐mediated delivery systems.

**FIGURE 13:**
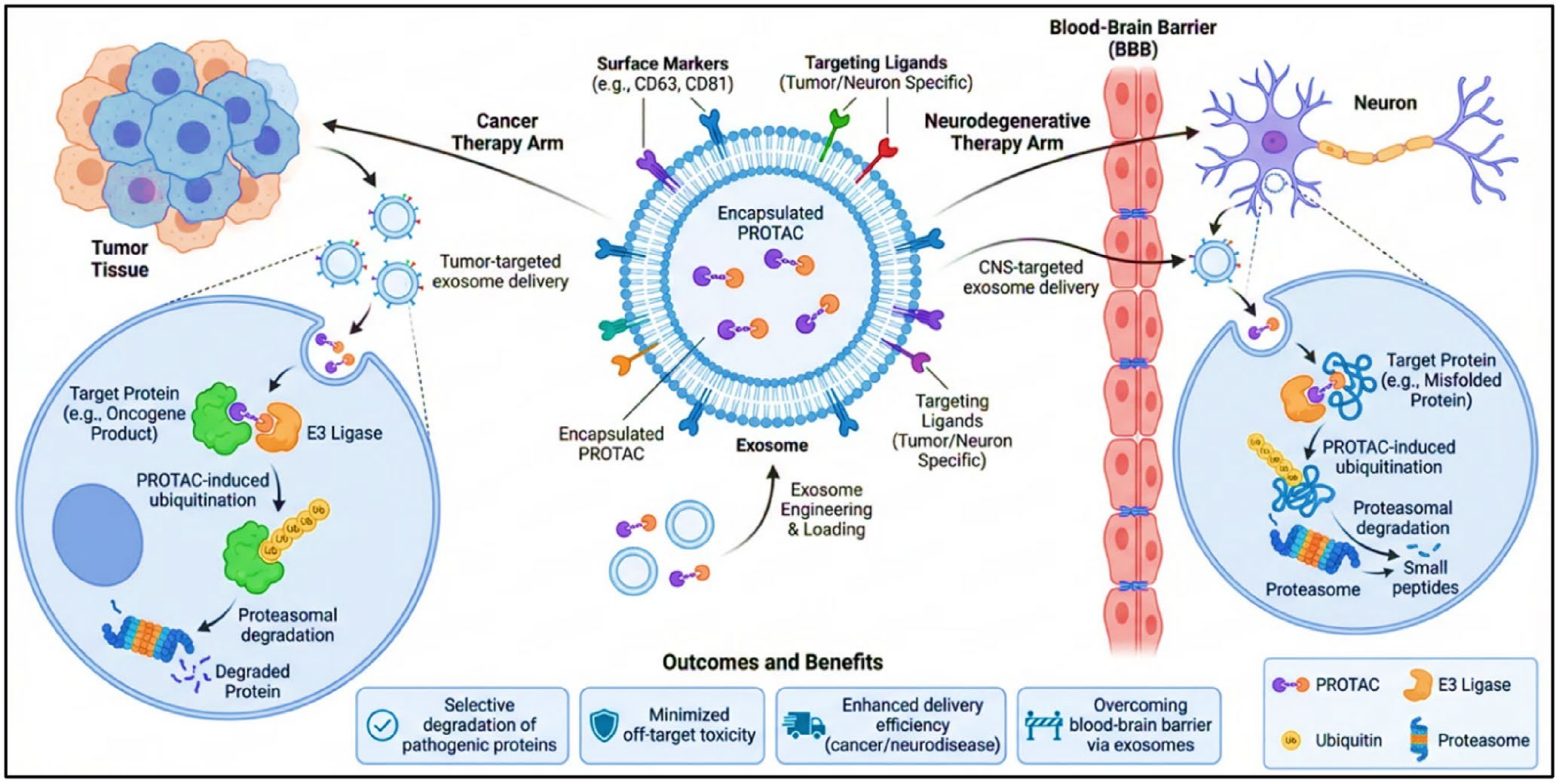
Future direction of research.

Exosome‐mediated PROTAC delivery remains at an early stage of development; it represents a compelling convergence of targeted protein degradation and biologically informed drug delivery. Addressing manufacturing, targeting, safety, and regulatory challenges through coordinated, multidisciplinary efforts will be key to establishing this platform as a viable and impactful component of next‐generation therapeutic strategies (Figure 13).

## Author Contributions

**Sruti Bagchi Ghosh:** conceptualization, investigation, software, methodology, validation, visualization, data curation, supervision, formal analysis, writing – original draft. **Tahani Ahmad ALMatrafi:** conceptualization, investigation, visualization, formal analysis, data curation, software, methodology. **Amany I. Almars:** data curation, supervision, conceptualization, methodology, validation, visualization, funding acquisition, writing – original draft, project administration, formal analysis. **Kranti Kiran Reddy Ealla:** conceptualization, writing – original draft, writing – review and editing, formal analysis, project administration, supervision, data curation. **Rumpa Banerjee:** investigation, conceptualization, writing – original draft, methodology, validation, visualization, software, formal analysis, supervision, data curation. **Fayez M. Saleh:** conceptualization, methodology, software, investigation, writing – review and editing, writing – original draft, visualization, validation, formal analysis. **Ajoy Kumer:** resources, writing – original draft, writing – review and editing, formal analysis, project administration, supervision, conceptualization, investigation, funding acquisition. **Bikram Dhara:** conceptualization, investigation, funding acquisition, writing – original draft, writing – review and editing, validation, visualization, project administration, formal analysis, supervision, resources. **Hailah M. Almohaimeed:** conceptualization, funding acquisition, writing – original draft, validation, visualization, project administration, data curation, software, methodology. **Zuhair M. Mohammedsaleh:** conceptualization, formal analysis, software, visualization, validation, methodology, writing – original draft, data curation.

## Funding

The authors have nothing to report.

## Conflicts of Interest

The authors declare no conflicts of interest.

## Data Availability

No new data was generated in this review.
